# Resilience of honeybee colonies via common stomach: A model of self-regulation of foraging

**DOI:** 10.1371/journal.pone.0188004

**Published:** 2017-11-21

**Authors:** Thomas Schmickl, Istvan Karsai

**Affiliations:** 1 Artificial Life Lab of the Department of Zoology, Karl-Franzens-University Graz, Graz, Austria; 2 Department of Biological Sciences, East Tennessee State University, Johnson City, TN, United States of America; Ghent University, BELGIUM

## Abstract

We propose a new regulation mechanism based on the idea of the “common stomach” to explain several aspects of the resilience and homeostatic regulation of honeybee colonies.

This mechanism exploits shared pools of substances (pollen, nectar, workers, brood) that modulate recruitment, abandonment and allocation patterns at the colony-level and enable bees to perform several survival strategies to cope with difficult circumstances: Lack of proteins leads to reduced feeding of young brood, to early capping of old brood and to regaining of already spent proteins through brood cannibalism. We modeled this system by linear interaction terms and mass-action law. To test the predictive power of the model of this regulatory mechanism we compared our model predictions to experimental data of several studies. These comparisons show that the proposed regulation mechanism can explain a variety of colony level behaviors. Detailed analysis of the model revealed that these mechanisms could explain the resilience, stability and self-regulation observed in honeybee colonies. We found that manipulation of material flow and applying sudden perturbations to colony stocks are quickly compensated by a resulting counter-acting shift in task selection. Selective analysis of feedback loops allowed us to discriminate the importance of different feedback loops in self-regulation of honeybee colonies. We stress that a network of simple proximate mechanisms can explain significant colony-level abilities that can also be seen as ultimate reasoning of the evolutionary trajectory of honeybees.

## Introduction

Efficient allocation of workers to appropriate tasks that ensure an adequate nutrient flow into the colony is a crucial challenge for insect societies. Colonies of wasps, ants, termites and honeybees commonly operate with lots of workers and have similar high numbers of brood in compound spaces like anthills, nests, or hives [1, 2]. To support the living expenses of these vast and dense colonies and to adapt to environmentally induced changes of the nutrient inflow, social insect colonies need operating mechanisms that allow a flexible regulation of an efficient supply of nutrients, oxygen and waste products.

The division of labor in honeybee colonies (*Apis mellifera* L.) is subject to an age-correlated regime (age polyethism) [3, 4], where workers determine their preferred engagements in specific tasks based on their age cohort. This worker allocation mechanism is reacting slowly to the environment and to changing colony needs, because under normal conditions the age distribution does not change rapidly and for recruiting new workers, the colony needs time for allowing the appropriate cohort to mature into the new worker roles. In fact, this slow age dependent task allocation mechanism is augmented by other mechanisms that allow faster reactions to environmental changes in which workers self-regulate the division of labor [2]. Besides the well-known dance regulation mechanisms [5–8], nurses were found to assess chemical stimuli about the hunger state of brood to determine whether they engage to the feeding task [9, 10]. In an ultimate reasoning, it can be assumed that natural selection has favored flexible self-regulatory proximate mechanisms that are scalable and robust enough to promote the survival and reproduction of colonies. For a colony, survival throughout cold winter periods requires accumulation of enough nutrients and workers, while reproductive success requires rapid growth in spring to allow reproductive colony division by building swarms [7].

Honeybees do not only stockpile nutrients, but they also have developed several regulation mechanisms to ensure efficient foraging and allocation of nutrients that function even in unfavorable foraging conditions (rain, wind, cold days). It was shown that pollen, the main protein reserve of honeybees, is accumulated at low storage levels compared to nectar/honey, thus it is available only as a short reserve lasting for a few days [11]. In a previous study, we analyzed that alternative mechanisms, not characteristic to honeybees, such as collecting and stockpiling more pollen in favorable weather (also called “pollen hoarding”), would have detrimental effect on colony success [12] when compared to a natural strategy that keeps low emergency reserves and fast reacting compensation mechanisms. In times of pollen shortage (caused by several days of rain or by a pollen trap installed by a beekeeper in front of the hive’s entrance), honeybee colonies perform several compensating strategies in parallel. Pollen foraging becomes significantly enhanced at the expense of nectar foraging [3], brood levels are reduced significantly [11], and feeding of brood is reduced [13, 14]. Cannibalism of eggs and larvae [15] enables re-allocation of proteins already spent on brood care [16, 17] in order to rear a few well-fed larvae [15] while keeping the food supply of the queen on an almost consistent level [14]. All of these compensating strategies require that individual workers change their behavioral patterns and adapt their task selection in short term (see for example [14, 18]. According to Lindauer [3] even very strong shifts in task selection concerning pollen foraging can happen within hours, at least within a day. The proximate mechanisms involved in protein regulation in a honeybee colony were reviewed in detail in [19].

Besides foraging for proteinaceous pollen the colony requires a rich influx, storing and processing of energy-rich sugars from nectar that is converted into honey. Recruitment for nectar foraging is performed by waggle and round dances [5–7], which establish self-enhancing positive feedback loops. These feedback loops are compensated by negative feedback imposed by food-storing bees, which are a limited resource, because it takes time to recruit additional storers through tremble dances after long search for a storer bee [8]. The time needed for recruiting storers correlates positively with the ratio of returning foragers to available storer bees [20]. These search times for storers correlate negatively with the availability of empty storing combs for nectar or honey, thus it correlates positively with the saturation of the colony with those substances [21]. Collecting of nectar is also linked to pollen foraging, because the foragers are recruited from the same pool of workers. Changing the pollen foraging strategy will also affect the amount of nectar ultimately collected [12].

One type of the ubiquitous regulation mechanisms for task allocation can be interpreted as a “common stomach” (sometimes also called “social crop”) which incorporates the idea of limited resources (nutrients, workers) that are required to be accessible for other workers to successfully start or finish a given task [22–29]. Such a common stomach system has to be available to all workers in a shared way and the substances have to spread (flow, diffuse) inside of the common stomach. In case of an already well filled reservoir, it becomes difficult for workers to add more to this reservoir, while retrieving the shared substance from the reservoir becomes easy and fast under these conditions. However, when the common stomach is rather empty, the storing of additional amounts becomes easy and the retrieving gets difficult. The concept of a “common stomach”-based self-regulation ability of a colony is that the recruitment and abandonment processes for a given task result in changes of the future content of the common stomach in a delayed manner. The status of the common stomach is modulating a set of positive and negative behavioral feedback loops that self-regulate not only the common stomach itself but also affect division of labor and nutrient flows.

In our present study, we develop a mathematical model to describe the dynamics of adult forager populations, of brood population, and of protein and sugar stocks in the hive. Our mathematical model based on the central assumption that common stomach regulation mechanisms are crucial to govern task allocation, foraging and brood nursing. Using the model we address the following questions:

1. Can the common stomach achieve homeostatic regulation of work force, food levels and brood production comparable to experimental findings?
2. Is the proposed regulation mechanism able to react to colony perturbations analogously to empirical studies?
3. What are the general properties of this common stomach regulation mechanism?
4. How can the model parameters affect the predicted colony-level regulation?
5. What are the scaling properties of the system?
6. How sensitive is the system to crucial parameter changes?
7. What are the contributions of the different feedback loops in the global regulation of the colony?

Our main hypothesis is that a network of feedback loops is regulating colony-level behaviors by modulating individual task selection in honeybees. Several feedback loops that regulate pollen and nectar foraging activities can be described (and thus modeled) by two “common stomachs”: The first common stomach is the saturation of workers and brood with proteins, affecting both, the influx and the consumption of pollen. The second common stomach is the saturation of nectar stores, which affects the ratio of loaded to unloaded storer bees, which in turn regulates the recruitment of nectar foraging bees. These two systems of feedback regulation interact with each other in the regulation of division of labor of foragers, as both forager groups (pollen foragers and nectar foragers), are drawn from the same limited pool of inactive potential forager bees. We hypothesize that this regulation system does not only affect work and nutrient stores in the colony, but also the brood age composition through cannibalism, what ultimately affects the age-polytheism on a longer time scale.

## Methods and models

### 2.1. Main model structure

**Fig 1:**
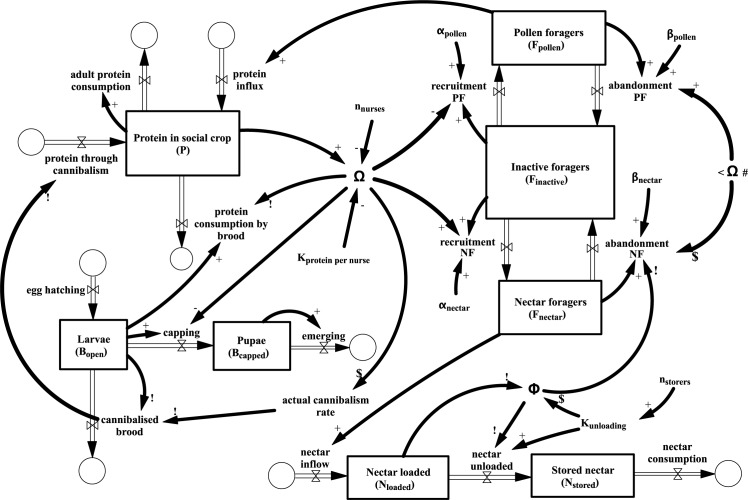
Stock&flow representation of our model system.

A general overview and the relationships of the main variables and feedback mechanisms are represented by the system dynamics Stock&Flow illustration [30] (Fig 1). The boxes represent stocks of important system variables such as worker group sizes, groups of specific developmental stages of brood and the levels of nutrient stocks. Double arrows represent flows (changes) between these stocks and single arrows symbolize directional information that effect other variables and serve as input into functions. The circular symbols indicate sources through which quantities of material enter the modeled system and sinks through which quantities of materials leave the system, thus these symbols indicate the system’s boundaries.

Our modeled system contains all important regulation loops discovered (see details at the corresponding equations). We emphasize two variables that are cornerstone components of most feedback loops: The two „common stomachs“: Φ(t) and Ω(t). These two system variables model the protein saturation of nurse bees (Ω(t)) and the remaining nectar unloading capability of the colony (Φ(t)). Both system components play important roles as an information center for individual bees and thus play central roles in the regulation of the global colony performance. Derivation of parameters and their values and dimensions are explained in the supplemental material (S1 File).

### 2.2. Model equations

The protein saturation of nurses is affecting the behavior of foragers in the decision whether or not to forage for pollen [31]. This regulation is assumed to happen through trophallactic feedings [32] of foragers by nurses. Thus, our first common stomach Ω(t) expresses this protein saturation of nurses

$$\Omega (t)=P(t)/(K_{protein\_per\_nurse}*n_{nurses})$$ (1)

where K_protein_per_nurse_ is the maximum protein that can saturate a nurse bees n_nurses._

Dreller and Tarpy [33] conducted a series of experiments that altered the brood-to-pollen ratios with occasional restrictions of bees by cages that restricted bees from entering specific hive areas but allowed olfactory cues to pass. They showed that the ratio of pollen foraging to nectar foraging activity was regulated according to the ratio of the number of accessible pollen cells to the number of larvae to be fed. This supply-to-demand ratio had a significant effect on the ratio of pollen foragers in the total group of foragers. In general, the majority of those bees, which do not forage for pollen, perform foraging for nectar [3], except in very hot conditions when bees forage for cooling water [34, 35]. For the sake of simplicity, we did not address this special case.

The second common stomach of our modeled system (Φ(t)) is expressed by the ratio between the amount of nectar to be unloaded from returning nectar foragers and the capacity for unloadings in the hive (Eq 2).

$$\Phi (t)=N_{loaded}(t)/(N_{loaded}(t)+K_{unloading}),$$ (2)

where K_unloading_ = n_storers_ * K_nectar_per_storer_. The parameter K_nectar_per_storer_ represents the amount of nectar that can be stored by every single storer bee in one hour. For building this model, we made the following assumptions: For a successful unloading, a returned nectar forager has to meet a storer bee [21]. We can consider these meetings of two bees like a random pick from an urn (having A’s and B’s). Each returned forager (A) randomly picks a bee and checks for being unloaded, what will happen only if the randomly picked bee is a storer bee (B). Thus the probability of a „successful”pick is p_(A picked by B)_ = B/(A+B) for every returned forager in the population (S1 File).

After modeling the common stomachs that govern the behavior of our system, we model the changes in worker groups engaged in the modeled tasks. First, the dynamics of pollen foragers are modeled as

$$\frac{dF_{pollen}}{dt}=\alpha_{pollen}*F_{inactive}(t)*(1-\Omega (t))-\beta_{pollen}*F_{pollen}(t)*\Omega (t),$$ (3)

where α_pollen_ and β_pollen_ represent recruitment rates and abandonment rates of the pollen foraging task. The process of recruitment for pollen foraging is modeled indirectly proportional to the common stomach variable Ω(t) and directly proportional to the available workforce F_inactive_(t). In contrast to that, the process of abandonment from pollen foraging is modeled directly proportional to Ω(t) and to the already recruited pollen foraging workforce.

The dynamics of the nectar foraging task are modeled as

$$\frac{dF_{nectar}}{dt}=\alpha_{nectar}*F_{inactive}(t)*\Omega (t)-\beta_{nectar}*F_{nectar}(t)*0.5*(1-\Omega (t)+\Phi (t)),$$ (4)

where α_nectar_ and β_nectar_ represent recruitment rates and abandonment rates of the nectar foraging task. The process of recruitment for nectar foraging is modeled directly proportional to the protein saturation of the colony (Ω(t)) while the process of abandonment from nectar foraging is modeled directly proportional to the already recruited nectar foragers multiplied by the average of the two common stomachs Φ(t) and Ω(t).

Finally, our model assumes conservation of mass of forager bees, as only open brood can die in our model. Thus, the number of inactive foragers per time step is modeled by

$$F_{inactive}(t)=n_{foragers}-F_{pollen}(t)-F_{nectar}(t),$$ (5)

where n_foragers_ is the total population of potential foraging bees (see S1 File).

**Fig 2:**
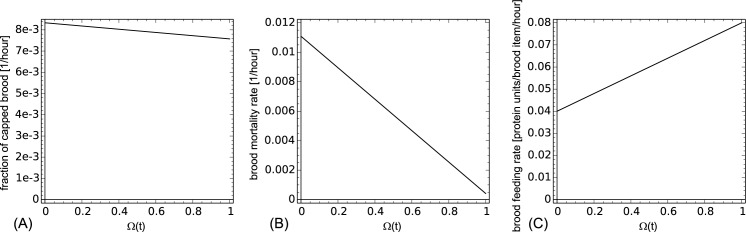
Model of brood capping (A), brood mortality (B) and brood protein feedings (C) depending on the saturation of the common stomach variable Ω(t). For details, see text.

In addition to adult forager bees, our model also predicts the dynamics of brood. For modeling these dynamics, it is important to consider that low protein supply also affects the mortality of larvae [15]. The rates of change of open brood (larvae, we neglect eggs here) are modeled by

$$\frac{dB_{open}}{dt}=\chi_{brood}-{(\lambda }_{hunger_{capping}}-(\lambda_{hunger_{capping}}-\lambda_{base_{capping}})*\Omega (t))*B_{open}(t)-(\mu_{base}+(\mu_{hunger}*(1-\Omega (t))))*B_{open}(t),$$ (6)

where χ_brood_ represents the rate at which eggs hatch into larvae. It was found that the capping rate depends on pollen (protein) supply, thus we scale the capping rate between λ_hunger_capping_ and λ_base_capping_ with increasing values of Ω(t) (Fig 2A). Similarly, larval mortality was found to increase significantly in times of insufficient protein (pollen) supply (S1 File, Fig 2B).

The dynamics of the capped (sealed) brood are determined by the already modeled rate of freshly capped larvae and by the hatching of old pupae to adults, what happens after approx. 12 days [36]. Our model explores short-term changes; thus we do not add these hatched pupae to the adult population. Analogously, we consider the adult population to be constant, thus we also do not consider adult mortality. The final dynamics of capped brood are modeled by

$$\frac{dB_{capped}}{dt}={(\lambda }_{hunger\_capping}-(\lambda_{hunger\_capping}-\lambda_{base\_capping})*\Omega (t))*B_{open}(t)-\lambda_{emerging}*B_{capped}(t),$$ (7)

where λ_emerging_ represents the pupal emerging rate. For our analysis of colony brood production efficiency, we accumulate all dead brood in an additional system variable B_dead_(t), which does not feed back into the model but allows us to keep track of the populations and to check for model consistency (conservation of mass).

After modeling the foraging workforce, the brood dynamics, and the pollen and nectar income, we can model the rates of change of the colony’s nutrient stocks. The changes in the shared protein stores are modeled by

$$\frac{dP}{dt}=\chi_{protein}*F_{pollen}(t)*\tau_{foragingfraction}-(\gamma_{hunger\_feeding}+(\gamma_{base\_feeding}-\gamma_{hunger\_feeding})*\Omega (t))*B_{open}(t)+(\mu_{base}+(\mu_{hunger}*(1-\Omega (t))))*B_{open}(t)*\chi_{cannibalism}-\lambda_{protein\_colony}*P(t)$$ (8)

where χ_protein_ represents the protein load collected by one pollen forager in one hour, τ_foragingfraction_ represents the fraction of day the bees are able to forage (due to the day-night cycle), γ_base_feeding_ represents the amount of proteins fed to a larva at times when protein reserves of the colony are maximized. The parameter γ_hunger_feeding_ represents the amount of proteins fed in times when the colony has minimum protein reserves, as such a reduced feeding rate was reported for low pollen periods by [13, 14]. The protein feedings of larvae are assumed to be depending on the common stomach Ω(t) (Fig 2 right panel).

Schmickl and Crailsheim [15, 19] as well as several older studies [16, 17] reported that honeybees may reuse a certain fraction of larval proteins that are gained by cannibalization of brood. The amount of cannibalized larvae is already modeled in the last term of Eq 6 as (*μ*_*base*_ + (*μ*_*hunger*_ * (1−*Ω*(*t*)))) * *B*_*open*_(*t*) thus we can simply multiply this amount with a protein-regain rate χ_cannibalism_, assuming that 50% of the total protein that is contained by one larva [37] can be re-utilized by the worker bees and that one larva is consumed within one hour. Finally, we assume a steady consumption rate λ_protein_colony_ for summing up all remaining, not yet explicitly modeled protein-consuming processes in the colony.

The dynamics of collected nectar brought to the colony by the foragers are modeled by

$$\frac{dN_{loaded}}{dt}=\chi_{nectar}*F_{nectar}(t)*\tau_{foragingfraction}-\lambda_{max\_unloading}*\phi (t)*K_{unloading},$$ (9)

where χ_nectar_ represents the amount of collected nectar per active nectar forager. For parameterizing this value, we considered the fact that nectar foragers return often partially loaded, as was reported by [38, 39]. The unloading of collected nectar is modeled as a fraction of the maximum unloading capacity (K_unloading_). It is modeled proportional to the common stomach Φ(t) and considers a maximum unloading interval of λ_max_unloading_.

The saturation of nectar-storing bees is a cornerstone element in the functioning of the whole system. When empty storer bees are scarcely available, the returning foragers will queue up for longer times and they will not be available for other tasks while waiting for unloading. This has a detrimental effect on the availability of workforce for other tasks. We considered three alternatives to model the unloading process (S1 File). Based on the most realistic saturation function (detailed as model 3 in S1 File), we can model the dynamics of the sealed nectar by

$$\frac{dN_{stored}}{dt}=\lambda_{max\;\_unloading}*\phi (t)*K_{unloading}-\lambda_{nectar\_colony}*N_{stored}(t)-\gamma_{foragers}*(F_{pollen}(t)+F_{nectar}(t))-\gamma_{nectar\_brood\_open}*B_{open}(t)-\gamma_{nectar\_brood\_capped}*B_{capped}(t),$$ (10)

where λ_nectar_colony_ represents the consumption of nectar by all colony members, γ_foragers_ represents an additional nectar consumption of active foragers, γ_nectar_brood_open_ models the nectar used for feeding and heating all open brood that is present in the colony and γ_nectar_brood_capped_ models the heating expenses of the colony invested into capped brood.

### 2.3. Solving the equations in simulations

All experimental runs shown in this article were performed by solving equations by 4th order Runge-Kutta method using the math tool SAGE [40]. We used a time step size of Δt = 0.5 hr. Visualization of data sets was done with mathplotlib 1.3.1 and computation of automated analysis was performed with python 2.7.8. Sensitivity analysis was performed with Vensim 6.3 PLE-PLUS-Academic [41] and Latin-Hypercube sampling.

## Results

### 3.1 Basic model runs with default parameters

**Fig 3:**
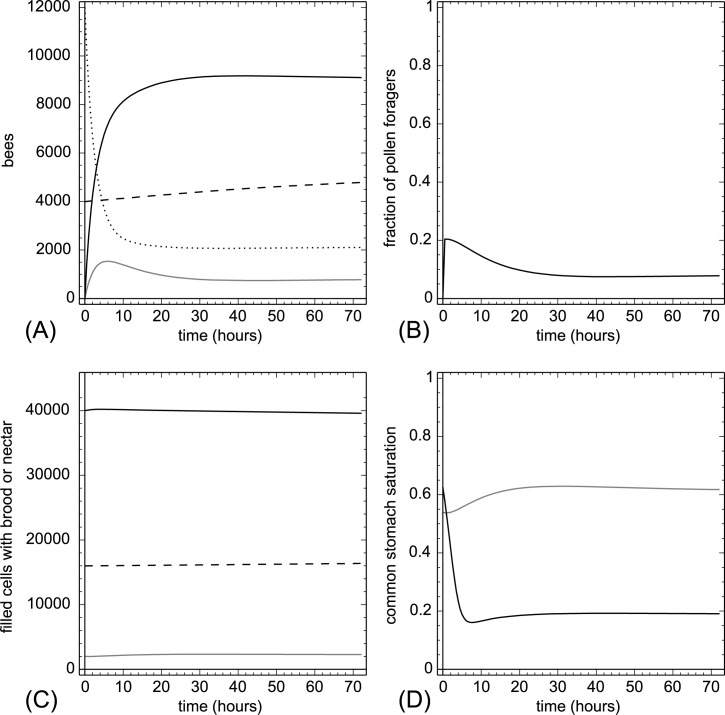
Undisturbed run with default model parameters (Table A in S1 File). A: dynamics of task groups and open brood: nectar foragers (black solid line), pollen foragers (gray solid line), inactive foragers (dotted line) and open brood items (dashed line). B: dynamics of the fraction of pollen foragers calculated as F_pollen_(t)/(F_pollen_(t)+F_nectar_(t)+1). C: dynamics of pollen stores (gray solid line), nectar stores (solid black line) and sum of open and capped brood (dashed line). D: dynamics of the common stomach saturation: Ω(t) (gray solid line) and Φ(t) (black solid line).

Running our model with our set of default parameters (S1 File) showed the intrinsic stability of the simulated honeybee colony in an undisturbed run: All system variables were holding equilibrium although some of them were started in extreme out-of-equilibrium conditions (e.g., all foragers started in the inactive state). Most inactive foragers converted quickly to nectar foragers and a small fraction (stabilized around 10%) into pollen foragers. There was a steady increase predicted for the number of open brood items while the total number of brood items, the number of nectar cells and the number of pollen cells stayed about the same. The two common stomachs stabilized on different levels (Fig 3).

### 3.2. Perturbations of the modeled system

After modeling the equilibrium conditions, we carried out several perturbation experiments that plausibly can happen to a honeybee colony to investigate the predicted responsiveness of the modeled colony. Our hypothesis is that the regulation by the two common stomachs Ω(t) and Φ(t) will homoeostatically regulate division of labor and nutrient supplies back to their set points or will let the colony approach a new stable equilibrium, thus will provide resilience for the colony system. In the following, we describe each perturbation experiment and specify detailed hypotheses about the common stomachs’ abilities to produce robustness, stability, yet also flexibility. In the following, we simulated a set of perturbation experiments that are classical in honeybee research.

#### 3.2.1. Modeling experimental removal and addition of resources, workers or brood

**Fig 4:**
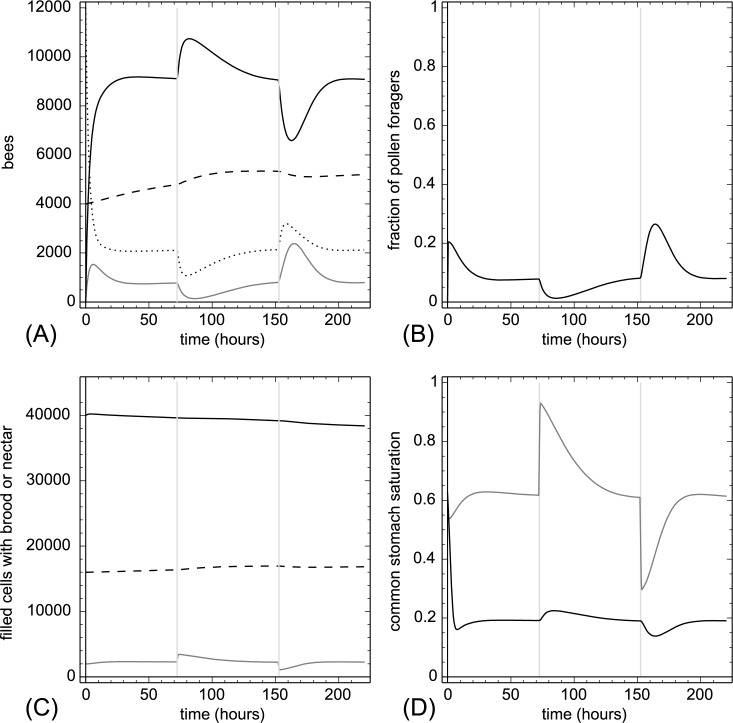
Pollen addition and removal. At t = 72 hr we added 20,000 punits of proteins and at t = 152 hr we removed 20,000 punits of proteins from the colony within one hour. The grey vertical lines indicate treatment periods. A: dynamics of task groups and open brood: nectar foragers (black solid line), pollen foragers (gray solid line), inactive foragers (dotted line) and open brood items (dashed line). B: dynamics of the fraction of pollen foragers calculated as F_pollen_(t)/(F_pollen_(t)+F_nectar_(t)+1). C: dynamics of pollen stores (gray solid line), nectar stores (solid black line) and sum of open and capped brood (dashed line). D: dynamics of the common stomach saturation: Ω(t) (gray solid line) and Φ(t) (black solid line).

All experiments were conducted with our default parameter set and default starting conditions (Table A in S1 File). After the colony has reached a stable equilibrium, adding or removing given quantities altered one worker group or one nutrient store. Whenever we removed workers of a specific working group, the population of total foragers decreased (n_foragers_) while all other worker groups remained constant (n_nurses_, n_storers_, n_others_). In case of removal of foragers, the equilibrium is expected to be at a lower level than before the disturbance, because the total workforce had decreased. We also hypothesize that the colony will compensate for sudden loss of nutrients. For example, pollen removal is hypothesized to increase pollen foraging by recruiting additional pollen foragers to replenish the pollen stores. The common stomach system provides a buffer for the colony that retains some supply with nutrients during the time needed to change the size of task groups in a compensatory reaction. Therefore, we hypothesize that the ultimate accumulation of nutrients (N_stored_(t)) and the overall production of brood (B_open_(t)+ B_capped_(t)) is only little affected by environmental disturbances, even if they affect crucial components like the protein influx through pollen foraging. We followed the changes of the main system variables during the following experiments:

**3.2.1.1. Pollen addition and removal.** At t = 72 hr we added 20,000 punits of proteins at once to P(t). This amount is equivalent to adding 1,176.5 pollen cells to the colony. At t = 152 hr we removed the same amount of protein again within one hour. Sudden addition or removal of proteins (usually done by adding or removing pollen-containing frames) is a severe disturbance of the colony. Our model showed very plausible responses (Fig 4). The experimental treatment resulted in a sudden strong change in Ω(t), which affected the recruitment of foragers quickly. Addition of pollen decreased the number of pollen foragers and increased the number of nectar foragers, while pollen removal had the opposite effect. While the total number of brood items was affected only moderately, the model predicted a significant increase in the number of larvae when pollen was added and a decrease when larvae were removed. Removing pollen also decreased the nectar stocks, because many nectar foragers converted to pollen foragers to compensate for the lost proteins.

**Fig 5:**
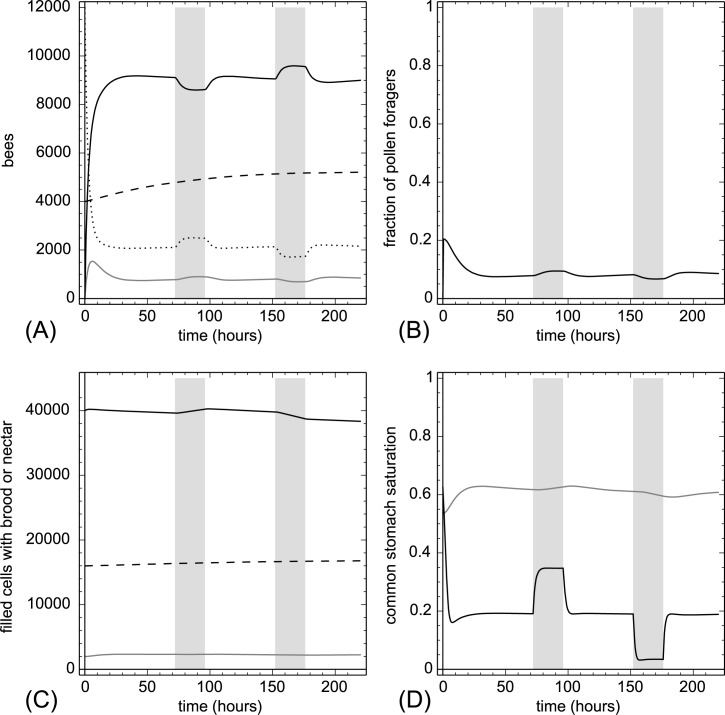
Nectar addition and removal. At t = 72 hr we added 1,000 nunits of nectar to N_loaded_(t)) in every hour for a period of 24h and at t = 152 hr we removed 1,000 nunits of nectar in the same way from N_loaded_(t)). The grey background indicates treatment periods. A: dynamics of task groups and open brood: nectar foragers (black solid line), pollen foragers (gray solid line), inactive foragers (dotted line) and open brood items (dashed line). B: dynamics of the fraction of pollen foragers calculated as F_pollen_(t)/(F_pollen_(t)+F_nectar_(t)+1). C: dynamics of pollen stores (gray solid line), nectar stores (solid black line) and sum of open and capped brood (dashed line). D: dynamics of the common stomach saturation: Ω(t) (gray solid line) and Φ(t) (black solid line).

**3.2.1.2. Nectar addition and removal.** Starting at t = 72 hr, we added 1,000 nunits of nectar per hour to the stock of unloaded nectar for a period of 24 hours. Starting at t = 152 hr, we hourly removed this amount of nectar again for a period of 24 hours. Adding and removing nectar stores strongly affected the nectar saturation Φ(t) of the colony and this in turn influenced the recruitment of foragers (Fig 5). Adding nectar decreased the number of nectar foragers and more pollen foragers were predicted to be recruited. Removing nectar had the opposite effect. These manipulations affected strongly the nectar stocks but did not considerably alter the pollen and brood stocks. However, the number of open brood items increased when nectar is added due to a resulting higher fraction of pollen foragers.

**Fig 6:**
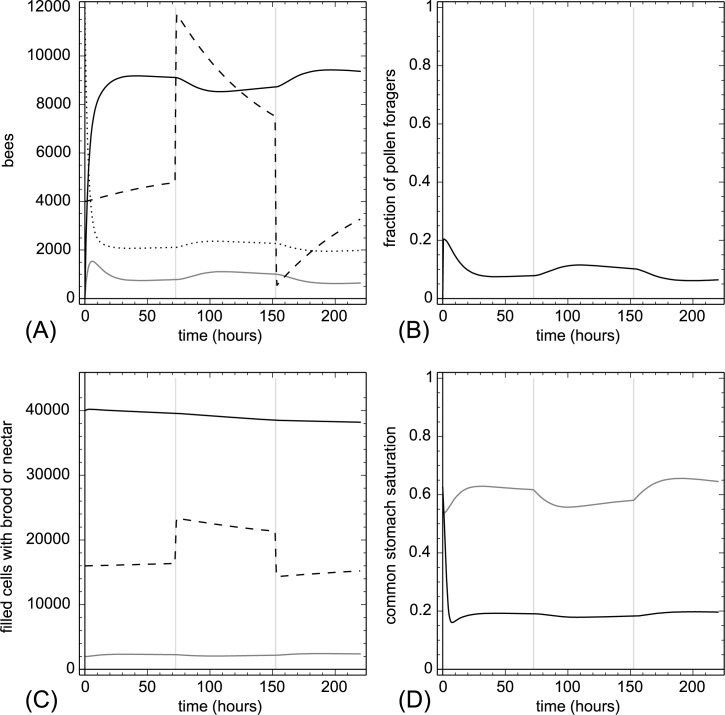
Brood addition and removal. At t = 72 hr we added 7,000 larvae (B_open_(t)) and at t = 152 hr we removed 7,000 larvae (B_open_(t)). The grey background indicates treatment periods. A: dynamics of task groups and open brood: nectar foragers (black solid line), pollen foragers (gray solid line), inactive foragers (dotted line) and open brood items (dashed line). B: dynamics of the fraction of pollen foragers calculated as F_pollen_(t)/(F_pollen_(t)+F_nectar_(t)+1). C: dynamics of pollen stores (gray solid line), nectar stores (solid black line) and sum of open and capped brood (dashed line). D: dynamics of the common stomach saturation: Ω(t) (gray solid line) and Φ(t) (black solid line).

**3.2.1.3. Brood addition and removal.** At t = 72 hr we added 7,000 larvae (B_open_(t)) and at t = 152 hr we removed the same amount of open brood again in one hour. Adding and removing open brood had only a weak effect on the nectar saturation Φ(t) but showed a strong effect on the protein saturation Ω(t) (Fig 6). Addition of larvae triggered higher recruitment of pollen foragers, however, as this took time, the pollen stores did still decrease and this in turn led to a loss of larvae (open brood) due to cannibalism. Removing larva had the opposite effect.

**Fig 7:**
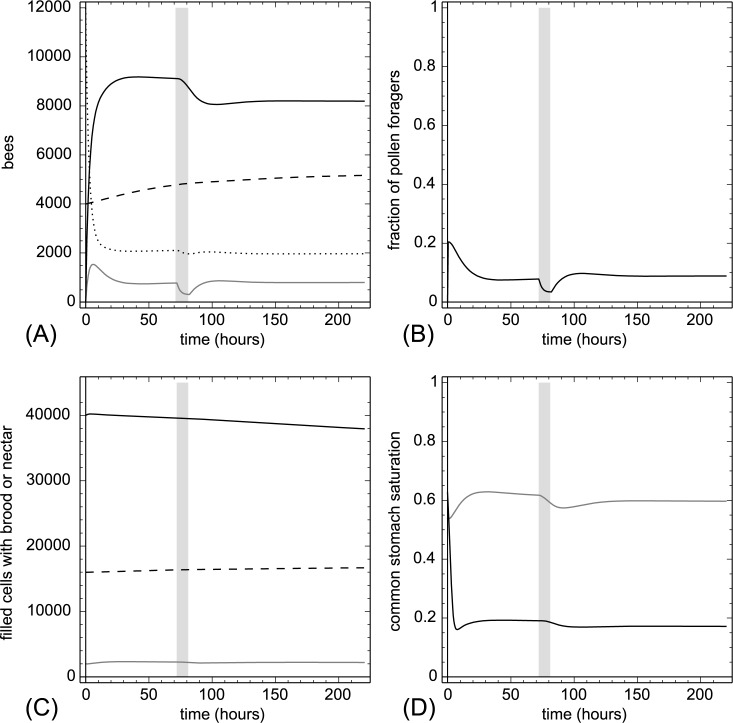
Removal of pollen foragers. At t = 72 hr we removed 25% of all pollen foragers (F_pollen_(t)) in every hour for a period of 10 hours. The grey background indicates treatment. A: dynamics of task groups and open brood: nectar foragers (black solid line), pollen foragers (gray solid line), inactive foragers (dotted line) and open brood items (dashed line). B: dynamics of the fraction of pollen foragers calculated as F_pollen_(t)/(F_pollen_(t)+F_nectar_(t)+1). C: dynamics of pollen stores (gray solid line), nectar stores (solid black line) and sum of open and capped brood (dashed line). D: dynamics of the common stomach saturation: Ω(t) (gray solid line) and Φ(t) (black solid line).

**3.2.1.4. Removal of pollen foragers.** Starting at t = 72 hr we removed 25% of all pollen foragers (F_pollen_(t)) in each hour for a period of 10 hours. In this experiment, we did not add back foragers to the modeled colony, because it is practically impossible to ensure that bees added to the colony would further act as pollen foragers. Experimental removal of pollen foragers decreased immediately the protein saturation Ω(t) and, in a delayed way, also decreased the nectar saturation Φ(t) of the colony (Fig 7). The loss of pollen foragers was well compensated after the removal period, but this happened at the cost of a resulting significant decrease of nectar foragers. This was because the total forager population had decreased after the removal of pollen foragers. This indirectly caused loss of nectar foragers in turn caused a significant decrease of the nectar stores, without significantly affecting the brood levels.

**Fig 8:**
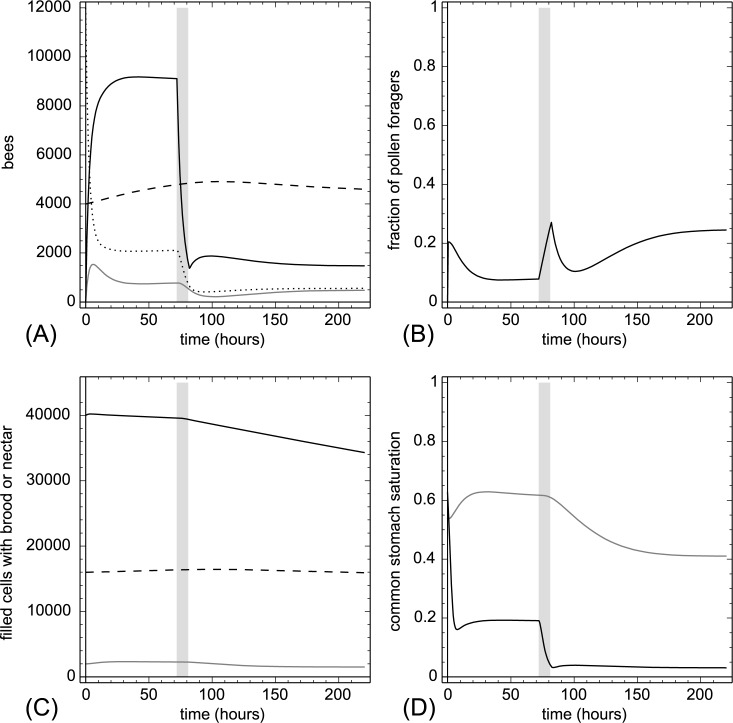
Removal of nectar foragers. At t = 72 hr we removed 25% of all nectar foragers (F_nectar_(t)) in every hour for a period of 10 hours. The grey background indicates treatment periods. A: dynamics of task groups and open brood: nectar foragers (black solid line), pollen foragers (gray solid line), inactive foragers (dotted line) and open brood items (dashed line). B: dynamics of the fraction of pollen foragers calculated as F_pollen_(t)/(F_pollen_(t)+F_nectar_(t)+1). C: dynamics of pollen stores (gray solid line), nectar stores (solid black line) and sum of open and capped brood (dashed line). D: dynamics of the common stomach saturation: Ω(t) (gray solid line) and Φ(t) (black solid line).

**3.2.1.5. Removal of nectar foragers.** Starting at t = 72 hr we removed 25% of all nectar foragers (F_nectar_(t)) in each hour for a period of 10 hours. In this experiment, we did not add back foragers to the modeled colony because it is practically impossible to ensure that bees added to the colony would further act as nectar foragers. Removing nectar foragers decreased immediately the nectar saturation Φ(t) and later also decreased the protein saturation Ω(t) of the of the colony (Fig 8). The colony only slightly compensated for the loss of nectar foragers after the removal period. Due to the considerable loss of the total forager population, the number of pollen foragers also decreased significantly. The removal of nectar foragers had only a very weak negative effect on the brood, but the nectar stores decreased significantly.

**Fig 9:**
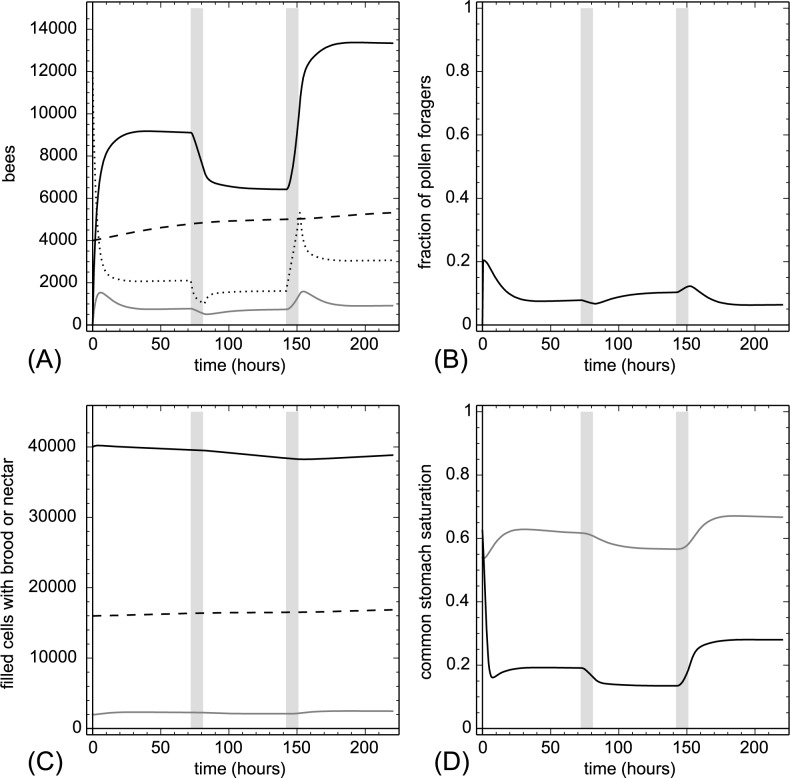
Addition and removal of inactive foragers. At t = 72 hr we removed 25% of all inactive foragers (F_inactive_(t)) in every hour for a period of 10 hours and at t = 152 hr we added 25% of all inactive foragers (F_inactive_(t)) to the bee population in every hour for a period of 10 hours. The grey background indicates treatment periods. A: dynamics of task groups and open brood: nectar foragers (black solid line), pollen foragers (gray solid line), inactive foragers (dotted line) and open brood items (dashed line). B: dynamics of the fraction of pollen foragers calculated as F_pollen_(t)/(F_pollen_(t)+F_nectar_(t)+1). C: dynamics of pollen stores (gray solid line), nectar stores (solid black line) and sum of open and capped brood (dashed line). D: dynamics of the common stomach saturation: Ω(t) (gray solid line) and Φ(t) (black solid line).

**3.2.1.6. Removal and addition of inactive foragers.** Starting at t = 72 hr we hourly removed 25% of all inactive foragers (F_inactive_(t)) for a period of 10 hours. Starting at t = 152 hr we added additional inactive bees by increasing F_inactive_(t) experimentally by 25% every hour for a period of 10 hours. Removing inactive foragers decreased the saturation levels of both common stomachs and this in turn decreased the size of both nectar and pollen forager populations (Fig 9). As a delayed effect, the pollen stores decreased significantly, but the effect on pollen stores and brood levels was not prominent. Adding inactive foragers to the system resulted in larger pollen and nectar forager populations and this in turn increased pollen and nectar stores and also the number of surviving brood. The saturation levels of both common stomachs also increased and this resulted in an even larger inactive forager population.

#### 3.2.2. Modeling rain periods and their effect on the colony

Rain periods can stop all foraging activities and this can last for long periods of time. The lack of foraging has not only profound consequences on nutrient inflows, but it can also affect division of labor and nutrient consumption in the hive. For simulating such rain periods, we set the values of recruitment rates (α) to 0.0 hr^-1^ and the values of the abandonment rates (β) to 1.0 hr^-1^ during the rain. This prevented the bees from foraging and turned active foragers into inactive foragers. In order to emulate the experimental procedure of a field experiment described in [11, 15], we simulated 5 days of rain after 6 days of no rain in a repeated pattern.

**Fig 10:**
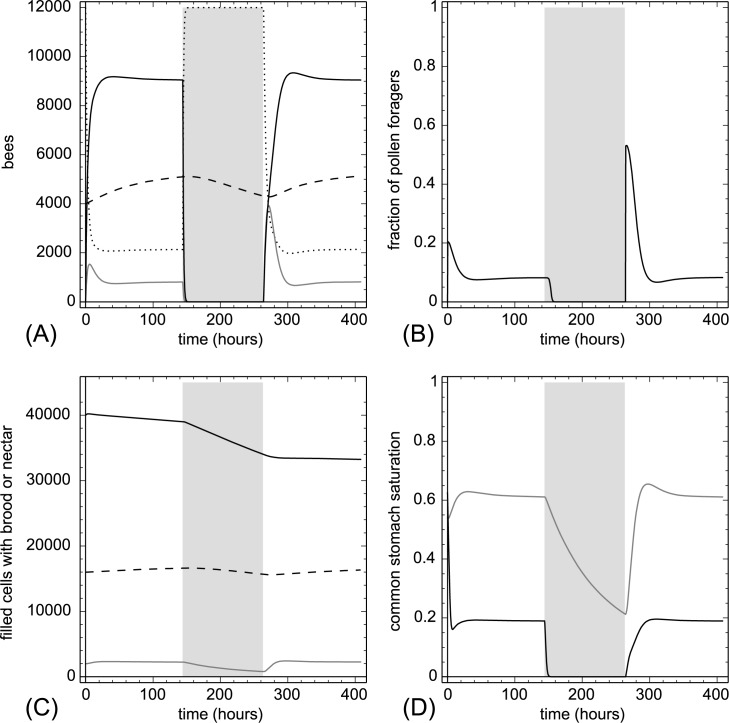
The effect of a rain period on the colony. After six days of standard conditions (Table A in S1 File) a rain period (from t = 144 hr to t = 264 hr) was simulated which reverts all foragers to inactive foragers. After the rain period the colony was simulated again with the standard parameters. The grey background indicates rain periods. A: dynamics of task groups and open brood: nectar foragers (black solid line), pollen foragers (gray solid line), inactive foragers (dotted line) and open brood items (dashed line). B: dynamics of the fraction of pollen foragers calculated as F_pollen_(t)/(F_pollen_(t)+F_nectar_(t)+1). C: dynamics of pollen stores (gray solid line), nectar stores (solid black line) and sum of open and capped brood (dashed line). D: dynamics of the common stomach saturation: Ω(t) (gray solid line) and Φ(t) (black solid line).

Our simulations showed that former active foragers quickly converted into inactive foragers during the rain (as β values are non-zero). The rapid loss of loaded nectar foragers quickly un-saturated the nectar-based common stomach Φ(t) (Fig 10). Larvae continued to consume the proteins derived from the non-replenished pollen stocks, in consequence, Ω(t) showed an exponential decay. Brood (especially open brood) decreased in number due to starvation and cannibalism. After the rain period, a peak in pollen foraging emerged, then the system rebounded to the same equilibrium as it was before the rain. However, a rain-induced loss in larvae and nectar reserve was predicted to be still observable in the following days. These system variables could rebound only after a long period of time.

#### 3.2.3. Modeling a pollen trap to prevent pollen foraging to a high extent

In contrast to rain periods, which prevent all types of foraging activities, a pollen trap specifically impairs the efficiency of pollen collection, as such a device removes most of the pollen loads from returning pollen foragers. It does not prevent the pollen foraging activity and has no significant effect on nectar collection. In apiculture pollen traps are commonly used to boost pollen foraging (hence pollination) activity [42]. Such a treatment significantly decreases the protein influx, but it does neither change the nectar influx nor does it affect the energetic costs associated with the foraging activity itself. To simulate such a pollen trap, we reduced the protein collected by every forager (X_protein_) from 1.0 punits/bee/hr to 0.05 punits/bee/hr, thus we assumed a 95% efficiency of the pollen trap. Emulating field experiments [15, 3] we simulated a 5 days long pollen trapping after 6 days of normal conditions and followed the effect of this treatment for 400 hours.

**Fig 11:**
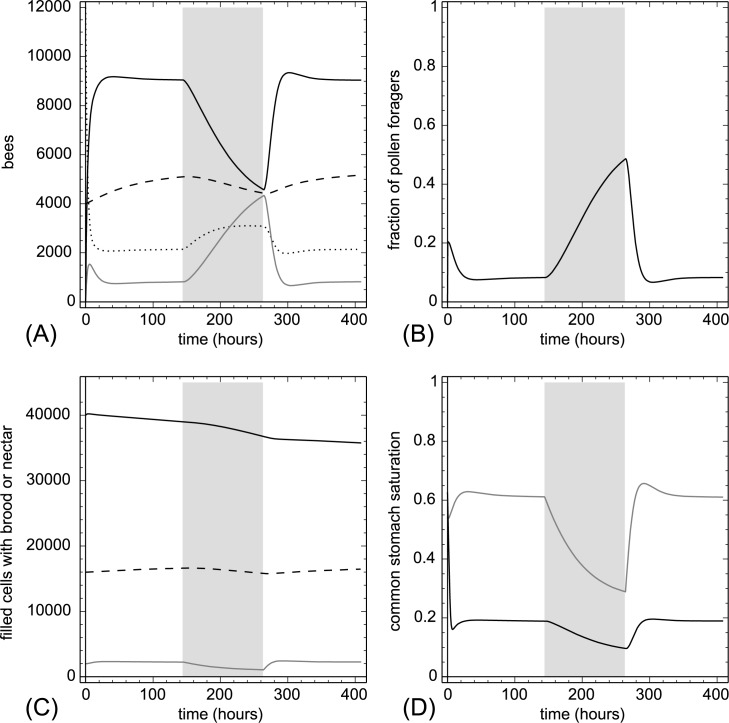
The effect of pollen trap on the colony. After six day of standard conditions (Table A in S1 File) a pollen trap was simulated with 95% efficiency (only 5% of the pollen reached the hive) from t = 144 hr to t = 264 hr. After the pollen trapping period the simulation was continued with the standard parameters. The grey background indicates pollen trap periods. A: dynamics of task groups and open brood: nectar foragers (black solid line), pollen foragers (gray solid line), inactive foragers (dotted line) and open brood items (dashed line). B: dynamics of the fraction of pollen foragers calculated as F_pollen_(t)/(F_pollen_(t)+F_nectar_(t)+1). C: dynamics of pollen stores (gray solid line), nectar stores (solid black line) and sum of open and capped brood (dashed line). D: dynamics of the common stomach saturation: Ω(t) (gray solid line) and Φ(t) (black solid line).

The pollen trapping caused an exponential decay in the pollen stocks and in the associated common stomach variable Ω(t) (Fig 11). This enhanced the recruitment of pollen foragers and their numbers increased by several fold at the cost of nectar foragers. This decline of the nectar foragers caused also a decrease of Φ(t) and of nectar reserves. Brood suffered heavy losses similar to the previous experiment (Fig 10). After the pollen trap was removed, the colony quickly rebounded with the exception of nectar reserves, which required a longer time to rebound.

### 3.3. Validating the model by comparing simulations to empirical experiments

We selected four historical experiments focusing on pollen trapping, pollen stores manipulation and manipulation of brood stores to be compared to the predictions of our model.

#### 3.3.1 Lindauer’s pollen-trap experiment (pollen trap only)

Martin Lindauer [3] reported an experiment in which a pollen trap was installed on 2^nd^ of May in an experimental colony, while a nearby control colony was kept without pollen trap. A pollen trap is a device that rips pollen loads from returning forager’s legs, thus it impairs the pollen foraging efficiency, but it does not prevent the foraging flights. The reported data starts 8 days after the installation of the pollen trap (10^th^ of May) and showed a daily recording of the fraction of pollen foragers in the total forager population until 21^st^ of May. To simulate this experiment with our model we started the colony with our default conditions (Table A in S1 File) and let it run for 6 days to reach equilibrium conditions. Then we modeled the insertion of a pollen trap with 97% efficiency in one run by setting X_protein_ from 1.0 to 0.03 punits/bee/hr for the time period of t ≥ 144 hr, while we kept our default parameters in the second (control) run.

**Fig 12:**
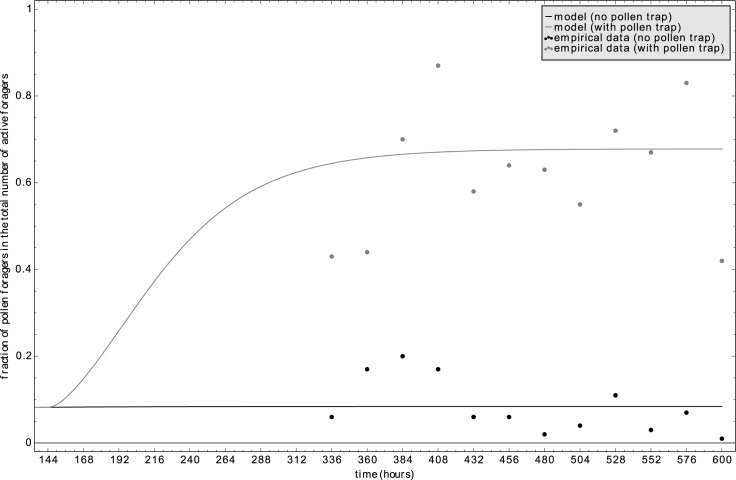
Simulation of Lindauer’s pollen trap experiment [3]. In the field experiment a control colony (black dots) and a colony with a pollen trap (grey dots) were compared by recording their fractions of pollen foragers 8 days after the pollen trap was applied. Our model prediction agreed with these data and demonstrated a seven-fold increase (grey line) from the control experiment (black line).

Installing a pollen trap significantly increased the ratio of pollen foragers in the overall foraging workforce (Fig 12). Protein saturation of the common stomach Ω(t) decreased with the artificial impairing of the pollen influx, because ongoing pollen consumption quickly depleted the pollen stores. The model predicted a change in the recruitment balance between pollen and nectar foragers, what fits well to the empirical data (Fig 12).

#### 3.3.2 Lindauer’s experiment with rain and pollen trap

Lindauer [3] also reported another experiment, which addressed the simultaneous effect of pollen traps and rain periods. He studied three honeybee colonies that were on normal conditions until 9^th^ of May. Then a rain period prevented the colony from foraging, which lasted until the 15^th^ of May intermitted with a short stop of raining on the 13^th^ of May. On the 19^th^ of May one colony was equipped with a pollen trap, while the other two colonies did not receive such a treatment. Thus, all three colonies first faced a foraging crisis due to the rain and in the second half of the experiment only one colony was stressed, while the other two colonies served as a control experiment. To reflect those experimental settings, we initialized our modeled colonies with our default parameter set (Table A in S1 File) 6 days before the first data collection to allow the colony to achieve homeostasis. The only divergence from these standard values was an increase of X_brood_ from 66 to 120 brood items [43] to fit our modeled colony to Lindauer’s colonies (full-sized colonies with high breeding activity). To simulate the rain periods, we set all recruitment rates of foraging to α_pollen_ = α_nectar_ = 0.0 hr^-1^ and all abandonment rates of foraging to β_pollen_ = β_nectar_ = 1.0 hr^-1^ for the time periods of 152 hr ≤ t ≤232 hr and 246 hr ≤at ≤ 300 hr. To simulate the pollen trap, we set the pollen foraging efficiency of pollen foragers (X_protein_) in one run from 1.0 to 0.03 punits/bee/hr for the time period t ≥ 388 hr to simulate a pollen trap efficiency of 97%.

**Fig 13:**
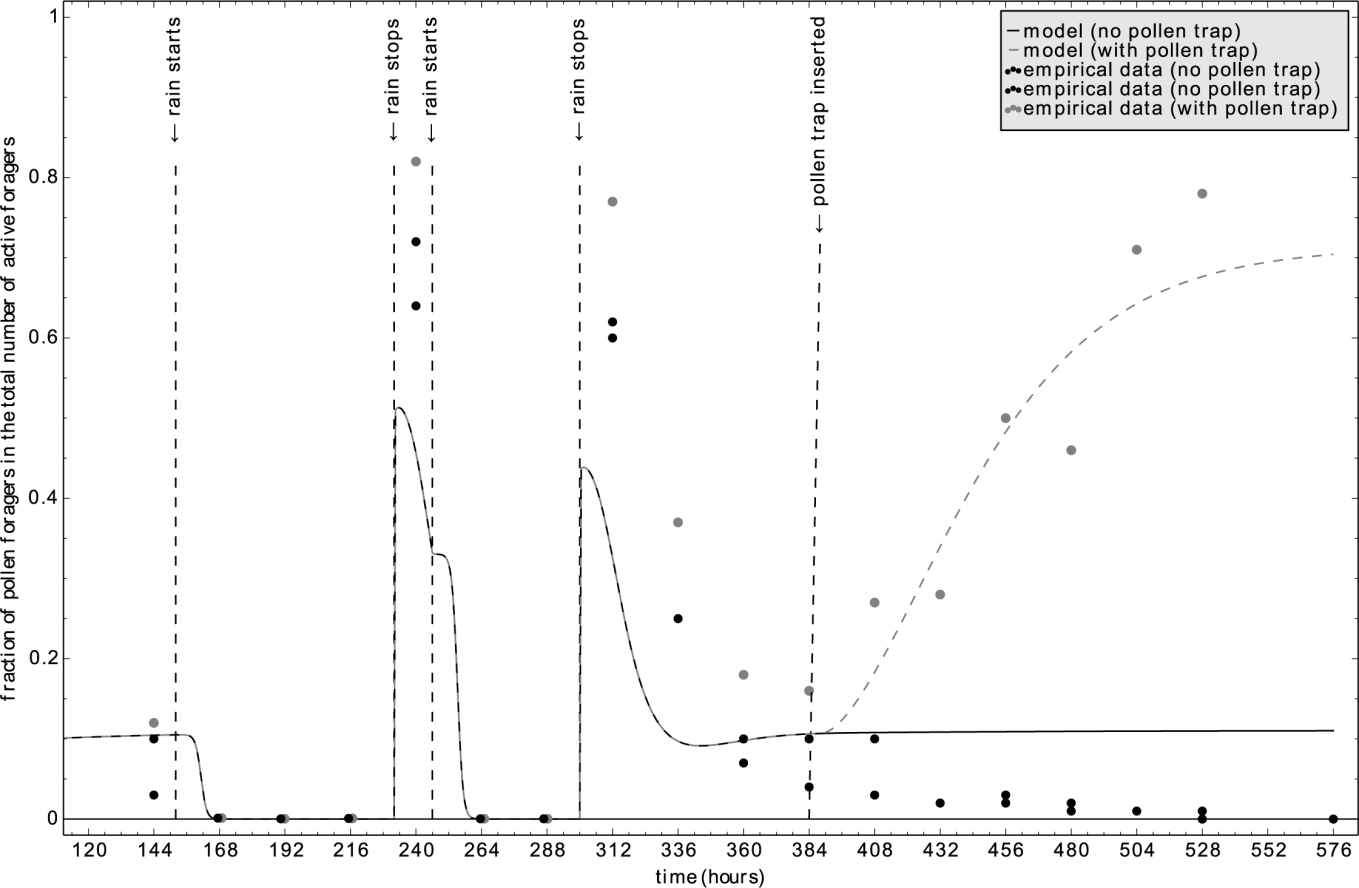
The effect of rain periods and pollen trap on the fraction of pollen foragers. The timing of experimental manipulations was following the empirical study. Redrawn measurements from Lindauer [3]: control: black dots; colony with pollen trap (gray dots). Predictions of the model: control: solid line; application of 97% efficient pollen trap: broken line.

Results of our simulations agreed with the data of Lindauer [3] and emulated well the effect of pollen trapping and rain (Fig 13). Before these perturbations each colony operated with a low fraction (around 10%) of pollen foragers. Rain periods prohibited foraging, but right after the rain the fraction of pollen foragers increased several folds and then went back to the normal level again. Introducing a pollen trap increased the fraction of pollen foragers and kept this value high.

#### 3.3.3 High and low pollen demand conditions

Rotjan et al. [44] individually marked 1,500 individuals of a honeybee colony of 16,000 bees, thus left 14,500 bees unmarked. Thus, we can define a normalizing factor of ω = 14,500/1,500 ≈ 9.66, assuming that whenever the experimenters identified a marked bee in performing a task it meant that in fact on average ω bees (1 marked bee and 9.66 unmarked bees) were performing this task. The authors established first low pollen demand conditions in the hive by adding pollen frames and removing brood frames. After 24 hours they altered the colony status to high pollen demand conditions by adding brood combs and removing pollen combs. They recorded the number of pollen flights on both days as well as the number of individual pollen foragers.

**Fig 14:**
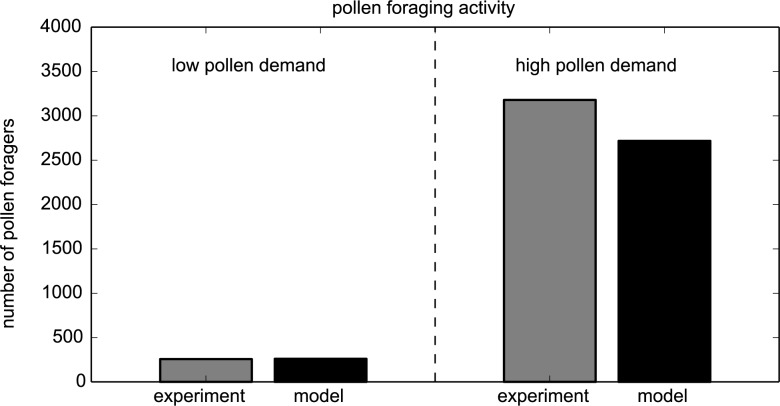
The effect of low and high pollen demand on the number of pollen foragers in a honeybee colony. Experimental data recalculated from [44] (grey columns) compared to the predictions of the model (black columns).

To conform our model to this field experiment, we started our model with our default parameter setting with some exceptions: We set the population to n_adults_ = 16,000 bees and reduced the egg laying activity to a marginal value of X_brood_ = 10 bees/hr. As indicated in the study, we started with very low brood populations of B_open_(0) = 25 larvae, B_capped_(0) = 80 pupae. The initial pollen stores were set to P(0) = 20,000 punits and the nectar stores to N_stored_(0) = 800,000 nunits. We gave the simulated colony 2 days to accommodate to equilibrium conditions. The recordings reported in the empirical study refer to t_1_ = 55 hr (1pm on day 1) and t_2_ = 79 hr (1pm on day 2) and the switch of colony pollen demand conditions was modeled to happen at t_3_ = 62 hr, corresponding to 8pm on day 1. To reflect the treatment, we removed pollen on day 2 from 62 hr ≤ t ≤ 64 hr by an extra outflow of 13,000 punits/hr and we simulated the extra brood gain on day 2 by setting the brood input X_brood_ (62 hr ≤ t ≤ 64 hr) by 3,000 larvae/hr. Using the ratio of marked and unmarked bees (ω), we estimated the total number of pollen foragers in colony of Rotjan et al. [44] to be 256 bees = 24*ω bees for low pollen demand and 3,179 bees = 298*ω bees for the high pollen demand case. Our model predicted the number of pollen foragers very closely to the experimental data (Fig 14).

#### 3.3.4 The effect of repeated rain periods on bee colonies

Blaschon et al. [11] artificially induced three five days long rain periods via a “rain machine” after 6 days of “good” weather (no rain conditions). The “rain machine” showered the hive entrance with cold water and casted shadow on the entrance. For our model, we used the standard parameters (Table A in S1 File) with few modifications, to conform better to Blaschon et al. [11] who used an observation hive instead of a full hive. Following the data of Schmickl et al. [14] for these hives, who observed the same colony as Blashon et al. [11], we set the colony to n_adults_ = 14,000 bees and set the egg laying rate to X_brood_ = 35 bees/hr. Blaschon et al. [11] reported the dynamics of pollen stores as well as the dynamics of brood older than 3 days, which we calculated from the amount of open brood in our model as follows: B_old_open_brood_(t) = B_open_(t)*0.3.

**Fig 15:**
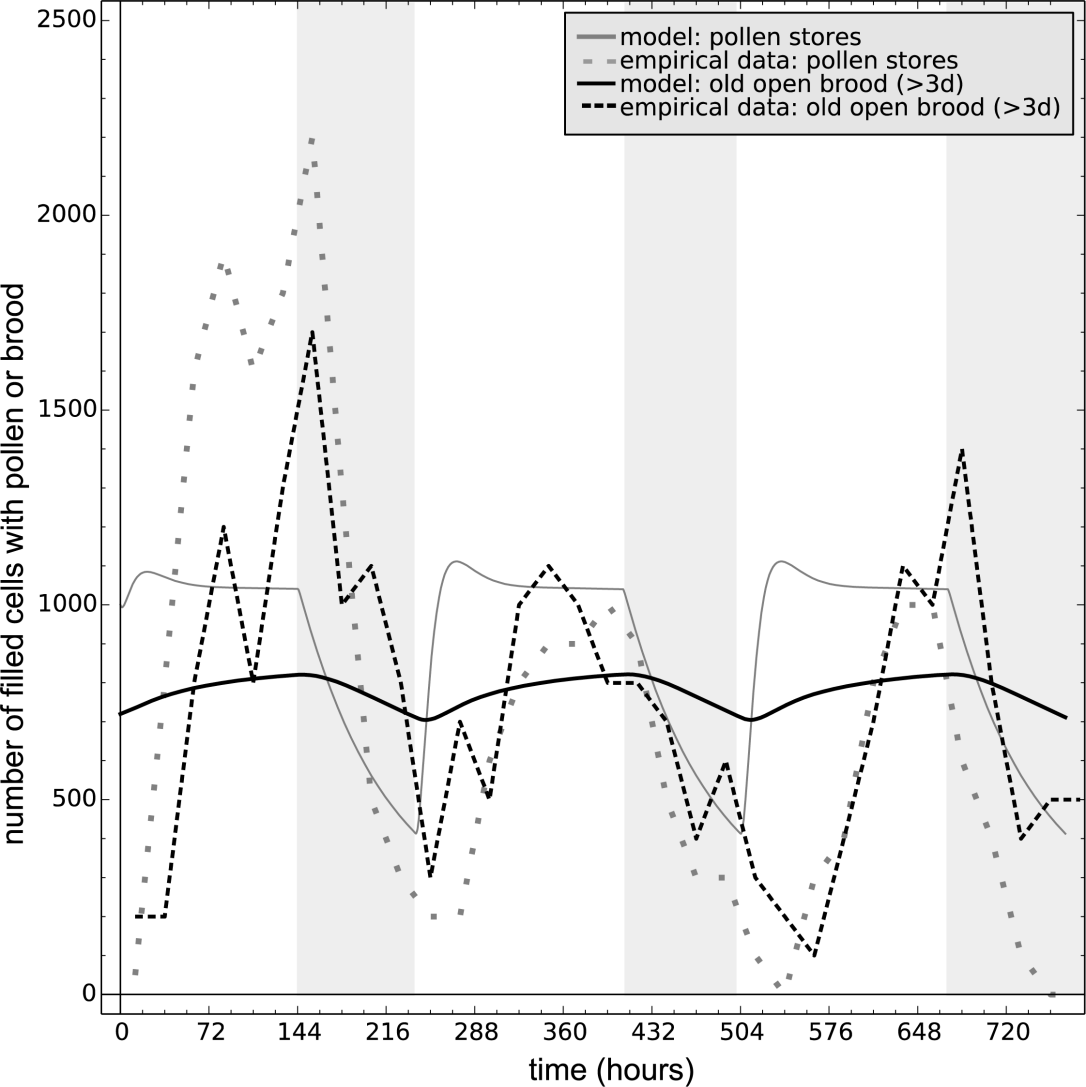
The effect of repeated rain periods on pollen stores and on old larvae. Experimental data redrawn from [11]: dashed line: open brood older than 3 days; dotted line: pollen stores. These data are compared with the prediction of the model: black line: old brood, grey line: pollen stores. Rain periods are indicated with grey background.

The prediction of our model followed qualitatively the observed dynamics of pollen and old open brood reported in the field study (Fig 15). Pollen stores decreased during the rain period, they showed a sudden increase just after the rain, and then they stabilized. The number of larvae older than 3 days also showed a decrease during the rain periods due to high cannibalism of younger brood. This cannibalism increased the loss of old brood in a delayed way [15]. One of the important novel predictions of our model is the discovery of the chain effect of “pollen → young brood → missing old brood”.

### 3.4. Testing the model’s sensitivity to parameter combinations

We derived each model parameter carefully from literature and logical deduction. Some of the parameters are invariable by definition, for example 1 punit is the amount of protein brought back to the hive by one active pollen forager and 1 nunit is the amount of nectar of a full cropload of one bee. Some other parameters are well supported from a biological perspective (e.g., the duration of the developmental stages of eggs, larvae, and pupae. Although the values assigned in our “default parameter set” (Table A in S1 File) are biologically plausible and were derived from literature, it has to be considered that many of the parameters can vary in nature. Thus, we needed to test that our model is not overly sensitive to such variations. The model can only be considered to be robust if it works plausibly within a possible range of key variables. In order to test whether or not our model’s predictions are stable against parameter changes, we varied all parameters listed in Table A in S1 File by ± 50% of their default value (Table 1). We conducted 10,000 independent experiments with randomized parameter sets within those given bounds by using Latin Hypercube Sampling in the sensitivity analysis tool provided by the modeling software VENSIM [41]. We recorded the dynamics of all major system variables in those runs: F_pollen_(t), F_inactive_(t), F_nectar_(t), B_open_(t), B_capped_(t), N_loaded_(t) and the two common stomach variables Φ(t) and Ω(t).

**Table 1:** Parameter range used for sensitivity analysis.

Analyzed variables	Range of tested values
β_pollen_	0.125–0.375
α_pollen_	0.075–0.225
β_nectar_	0.125–0.375
α_nectar_	0.250–0.750
χ_nectar_	0.126–0.378
λ_nectar_colony_	0.0005–0.0015
γ_nectar_brood_open_	0.01–0.03
γ_nectar_brood_capped_	0.0025–0.0075
γ_forager_	0.0105–0.0315
γ_hunger_feeding_	0.02–0.06
γ_base_feeding_	0.04–0.12
μ_base_	0.00021–0.00063
μ_hunger_	0.00533–0.0160
χ_cannibalism_	2.4165–7.2495
λ_protein_colony_	0.0025–0.0075
χ_brood_	33–99

To further test the stability of the system, we conducted a series of perturbations in each run:

- From t = 50 hr to 98 hr (PT): a pollen trap was installed with 99% efficiency (X_protein_ = 0.01 punits/bee/hr).
- From t = 150 hr to 151 hr (P-): 10,000 punits were removed from P(t).
- From t = 200 hr to 201 hr (P+): 10,000 punits were added to P(t).
- From t = 300 hr to 301 hr (BO+): 3,500 larvae were added to B_open_(t).
- From t = 350 hr to 351 hr (BO-): 3,500 larvae were removed from B_open_(t).
- From t = 400 hr to 412 hr (N+): 400 nunits were added to N_loaded_(t) each hour.
- From t = 450 hr to 462 hr (N-): 400 nunits were removed from N_loaded_(t) each hour.
- From t = 500 hr to 510 hr (FP-): 25% of pollen foragers were removed from F_pollen_(t) each hour.
- From t = 550 hr to 560 hr (FI+): 250 inactive foragers were added to F_inactive_(t) each hour.
- From t = 600 hr to 610 hr (FI-): 250 inactive foragers were removed from F_inactive_(t) each hour.
- From t = 650 hr to 660 hr (FN-): 25% of nectar foragers were removed from F_nectar_(t) each hour.

**Fig 16:**
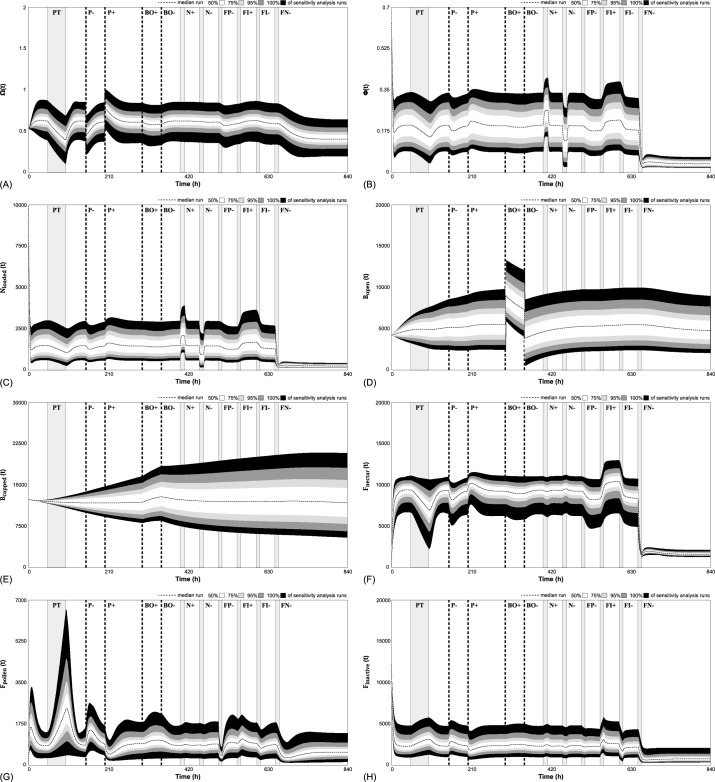
Sensitivity analysis of major system variables Ω(t), Φ(t), and our major stock variables N_loaded_(t), B_open_(t), B_capped_(t), F_nectar_(t), F_pollen_(t), and F_inactive_(t). The dotted lines represent the median values of all runs and bands indicate the deviations of runs from these medians (percentiles and quantiles) of runs: white: 50%, light gray: 75%, dark grey 95% and black 100% of all 10,000 runs. See text for the timings and the actual perturbations of the experiments (two-letter codes with “+” or “-”for indicating addition and removal treatments).

The outcome of this sensitivity analysis (Fig 16) showed that our model is very stable and various combinations of different parameter values did not lead to implausible results in the major system variables even if parameter variations were paired with strong experimental perturbations. In most cases, the observed changes in these variables were clearly under-proportional to the induced parameter changes. None of the values became negative or showed an unbounded increase.

### 3.5. The importance of feedback loops that are modulated by the common stomachs

Our model is based on several feedback mechanisms, which regulate foraging and division of labor. The most prominent feedback loops involving the common stomachs are those that modulate recruitment and abandonment from the two foraging tasks. Besides these feedbacks, the common stomach Ω(t) is also involved in three other feedback loops that modulate brood caring activities of worker bees. To investigate the significance of these mechanisms, we performed a series of simulation runs in which one or more of these mechanisms were switched off (indicated by a “-“) or switched on (indicated by a “+”) while the model colony was facing a period of strong pollen stress imposed by a pollen trap:

1. Switching off the early capping in times of protein stress (“C-“): By setting λ_hunger_capping_ = λ_base_capping_we kept the capping rate constant in all conditions.
2. Switching off the reduced feeding of larvae in times of hunger stress (“F-“): By setting γ_hunger_feeding_ = γ_base_feeding_ we kept the feeding rate constant in all conditions.
3. Switching off protein regain through (“R-“): By setting X_cannibalism_ = 0.0 punits/larva we prevented any protein regain through cannibalism.

In all runs, we started the simulated colony with our default parameter set and let it run for 48 hr to allow it to achieve equilibrium. At t = 48 hr we simulated a pollen trap with 99% efficiency by setting X_protein_ = 0.01 punits/bee/hr.

**Fig 17:**
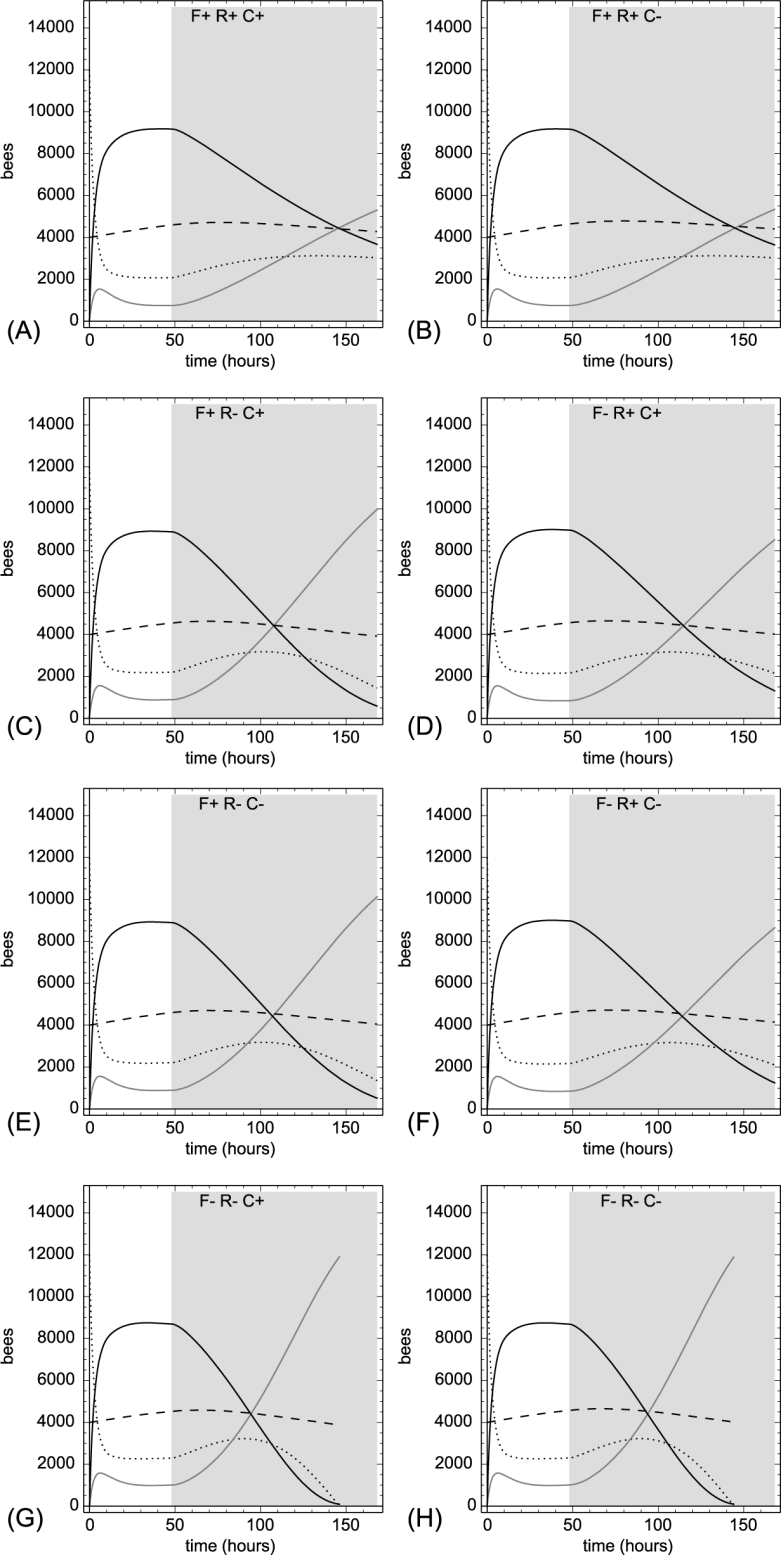
The effect of feeding reduction (F), protein regain from cannibalism (R) and early capping of hungry brood (C) on foraging and brood. Active feedback is indicated by a “+” symbol and a deactivation of a feedback loop is indicated by a “-”symbol. After running 48 hours with standard parameters a pollen trap with 99% efficiency was introduced (grey background). Solid black line: nectar foragers; Solid grey line: pollen foragers; Dotted line: inactive foragers; Dashed line: brood).

When we kept every feedback loop active (Fig 17A, F+R+C+), the model colony reacted to the pollen trap similarly as in our other pollen trap experiments: The number of pollen foragers increased, the number of nectar foragers decreased significantly. The brood decreased only slightly due to the shortage in pollen income. The deactivation of the cannibalism feedback changed the results only marginally (Fig 17B, F+R+C-), but selective deactivation of feeding reduction (F) and protein regain from cannibalism (R) had a stronger effect (Fig 17C and 17D, F+R-C+, F- R+C+). In these two cases, the effect of the pollen trap was significantly stronger on the colony: The pollen-to-nectar-forager ratio was shifted significantly more towards the pollen foraging, and less brood survived. In all three cases in which we deactivated only one feedback loop the system was still performing rather well under stress.

When we deactivated more than one feedback loop at once, the system reacted even stronger and the negative effects on the colony (e.g., brood loss, low nectar foraging) were significant (Fig 17E and 17F, F-R+C-, F+R-C-). In two cases, when both feeding reduction (F) and protein regain from cannibalism (R) was switched off (Fig 17G and 17H, F-R-C+, F-R-C-), the system collapsed before the 150^th^ hour, because all foragers had already turned into pollen foragers, yet they were still unable to provide enough pollen (Ω(t) was approaching zero). These experiments showed that the modeled regulatory mechanisms are important for stabilizing the system in addition to the main feedback loops that are linked primarily to foraging recruitments. These results also indicated that the protein-based common stomach Ω(t) regulation network was more complex (e.g., involved in more important feedback loops) and more important than the nectar regulating common stomach Φ(t).

## Discussion

Honeybee colonies are exceptionally robust and resilient societies. Such resilience is characterized by a system’s ability of exceeding or returning to a level of performance it had before a disturbance. The system remains functional while it may change its state in response to the disturbance [45]. The nature of resilience of honeybee colonies cannot be explained solely by their main division of labor mechanism, which is age polyethism, because it could not explain the observed flexibility, if it was acting alone. To mention one example, hastening biological development in honeybees can lead to colony collapse, because young foragers lack experience and are thus inefficient [46]. However, other studies showed that the usual age polyethism regime can be altered in case of strong disturbances in age structure, for example the foraging onset can be postponed or nursing activities may be extended if colony age demography requires this [47, 48]. Similar results were reported by Wilson [49] for division of labor in ant colonies. We can assume that multiple mechanisms are working in parallel, which differ in their degree of flexibility. Some studies reported that individual honeybees lack flexibility in several tasks [50, 51]. However, regarding other tasks, like food storing, selection of foraged materials, or nectar source selection, a high individual worker flexibility was observed [21, 52]. These seemingly contradictory findings indicate that a flexible colony level functioning requires regulation on two levels: Feedback loops on the individual behavioral level and on the group level. Our model considers both, as it assumes that individual bees can switch between two tasks and this recruits worker cohorts to group sizes that fulfill adequately the colony needs.

The model links to age polyethism by predicting the brood dynamics and it links to colony-level fitness by predicting the dynamics of nutrient stores. This way the model “sits” exactly between models that predict population dynamics on a long-term [37, 53, 54] and models that depict behavioral foraging, storing decisions and nursing decisions on short time-scales [52, 55–59].

In the heart of the short-term regulatory mechanisms we propose, that there is a shared information center which we call “common stomach”. The regulation system using common stomach was found also in wasps [22–26]) and ants [28], therefore it is not surprising that bees can also rely on such an information center. A common stomach reminds in many aspects to the social crop [60, 61]: It represents a system in which quantities of material have both a lower and an upper bound and the consequence of this is a saturation of quantity between these bounds. This saturation regulates task recruitment that in turn can affect supply and demand concerning those quantities. Wasps store water for nest construction in their crops, but honeybee workers store nectar and pollen in wax combs, therefore we consider parts of the common stomach to be external. We stress that the saturation level of the common stomach governs the collection and use of the same material it stores through a network of feedback loops. The role of the common stomach is two-fold: (1) accessing it as an information center allows adjustment of the workforce in a colony-level homeostatic way; (2) it serves as a strong buffer of materials [29], therefore it absorbs fluctuations without the need of continuously readjusting the workforce balance, what would be inefficient and thus energetically costly [27].

Our model is focused on short time-scale regulation and on just some aspects of the division of labor found in bee colonies. Besides the pollen foraging, nectar foraging and inactive foragers, we omitted other tasks that are also regulated in honeybee colonies [62]. This decision was made not only for the sake of staying focused but also because of the fact that these aspects of colony integration were already extensively modeled in the past [52, 55, 56, 59, 63–69]. The regulation of pollen storing and nectar foraging workforce, which we addressed here, is less known than the regulatory role of dances, but these regulations also have serious consequences for colony survival and success [12]. Here we stress that the general understanding of these processes is highly relevant from evolutionary and ecological perspectives.

Our model depicts a honeybee colony with reasonable equilibrium values for worker distribution over tasks, brood states and nutrient stores (see Fig 3, answers to research question 1 and 3).

Our simulated experiments showed very plausible and reasonable predictions similar to the responses of honeybee colonies, namely, the colony either returns to the old equilibrium, or, in case the treatment changed the number of working bees permanently, to a new stable equilibrium (Figs 4–11, answers to research question 2). Changing the overall population size of the brood or workers did not impair the predicted ability to achieve a stable distribution of work. The colonies exhibited strong resilience even in case of significant losses and the workforce reorganized itself into a new level that allowed high brood survival and resource accumulation under these altered conditions (Figs 6–9, answers to research question 5). Detrimental effects of pollen traps or rain periods impeded the fulfillment of colony needs, but the colony was able to compensate these effects by shifting the workforce quickly to a new equilibrium (Figs 11–13, answer to research question 4).

When we compared our model predictions to experimental results reported in literature, we found remarkable qualitative validation of our model. This is not only answering our research question 2, but also strengthens our confidence in our model, as we never did structural change of the model itself, as always the same equations were used to simulate these experiments. We only had to set specific starting values for our stock variables and sometimes adjusted some specific parameter values to conform the experimental conditions, for example by setting the foraging recruitment rates to zero during the rain periods. Our main goal was to show that all special cases we studied can be derived from the same underlying mechanism we propose here with our model.

The sensitivity of the model to specific parameter changes revealed a striking stability of the model against such parameter changes (Fig 15, answer to research question 6 These sensitivity runs were carried out in extensive long-term simulation runs that incorporated all colony perturbations we had applied beforehand in our study. This stability treatment was shaking the system in two ways in many combinations. It showed that the model exhibits a high stability under all parameter values and a high resilience against all tested perturbations.

The regulation of the nutrition inflow is a complex network of feedback mechanisms. In the center of these regulations are the two common stomachs Φ(t) and Ω(t). These feedback loops primarily affect the recruitment of foragers to either nectar or pollen foraging. In addition to these feedback loops, several additional feedback mechanisms relating to protein saturation play important roles in brood care: protein regain through cannibalism, feeding reduction of brood and early capping. By selectively disabling these feedback loops we were able to assess their importance for the colony regulation (answer to our final research question 7). These mechanisms together provide a redundancy for stabilizing the colony. Disabling any one of these loops individually resulted in stable colonies that still work well. When two feedback mechanisms were disabled, the system was still able to cope with strong perturbations in two out of three cases. This analysis revealed that “regain of protein through cannibalism” was the most important feedback loop in the system in times of protein stress, the “feeding reduction of brood” mechanism provided the second-most important feedback loop, and the “early capping” mechanism was the least important feedback of those three. We analyzed the stability of the system concerning the protein regulation only, because the pollen stores in the hive are usually very small compared to nectar and honey stores, and they can change considerably during the range we simulated our model.

This stability of the model we described here is not only stemming from biological phenomena we considered, but also from the strict adherence to the 6 model building principles we outline in the supplementary document (S1 File). Our goal was not to build a model that fits specifically into a specific set of experimental data, but to construct a model system, which is in itself robust, resilient and still flexible and which predicts the general behavior of a honeybee colony. Our model parameters were carefully chosen to be either measured from empirical findings or deducted from logical reasoning from several data sources. Still, the model is not very sensitive to the exact value of those parameters, because the processes we implemented are robust. The regulation system we modeled is based on shared substances that can only be locally accessed for exploitation. Thus, the more scarce a substance is, the longer it takes for a worker to locate it and the slower the exploitation process gets. This protects the colony from overexploitation and gives additional stability to the system. All individual interactions were assumed to be linear and simple and this provided a very general model that could simulate a variety of experiments without requiring structural adaptation of the model itself.

Our results showed that the proposed regulation mechanisms are not only robust against perturbations but also insensitive against variation, but also show low parameter sensitivity. Such a low sensitivity of the mechanism to parameter variations indicates that these mechanisms are robustly working under a large variety of conditions (speeds of bees, load distributions of bees, brood levels, behavioral threshold values, etc.). This robustness is another potential advantage in natural selection of the mechanism proposed by our model. Finally, selective analysis of feedback loops allowed us to discriminate these feedbacks as well as the two common stomachs concerning their importance for honeybee self-regulation and resilience: We clearly indicated the adaptive significance of the existence of those mechanisms, which all involve the proposed common stomach regulation mechanism.

In the past, various mechanisms have been proposed for task regulation in social insects, for example reviewed in [70, 71] for various social or eusocial animals, and in more detail in [72] specifically for honeybees. Most of the work done so far in this field was based on mechanisms that basically build on top of positive feedback that rips a former rather evenly distributed set of agents into distinct sets of task groups or spatially located groups by amplifying random noise in start conditions or applied at runtime. This was shown in well-accepted mechanisms on ant pheromone trails [73–77] or food source selection [52, 66] and various organisms’ aggregation behaviors [70, 78, 79] in which the term “nonlinearity” is usually attributed to a strong positive feedback in the system. The mechanisms we propose here are emphasizing strong negative feedbacks in regulating task selection. Still the system is very reactive and able to cope with changing demands and perturbations. Similar reactive, resilient models that emphasized the role of negative feedback are known for spatial choices in ants [80, 81], for shelter choice in cockroaches [82] and for collective decision-making in fish [83] and especially for task regulation of *Metapolybia* wasps [22–26]. In young honeybees (before the forager age) such negative feedback was shown experimentally in [84], and then modeled algorithmically in [85–88]. Also, short term adaptation of the age-polyethism regime in honeybees was demonstrated in honeybees [89] yielding a pheromone-based social-inhibition model [90, 91]. Such a pheromone-inhibition system shows similarity to the common stomach model because it is based on negative feedback loops in which the shared substance is a pheromone and not a nutrient substance like in the study presented here. We want to stress that resilience and stability ensured by the regulatory role of strong negative feedback is a key element of the decentralized control of division of labor [45]. It ensures that the system is buffered against small changes [24, 29] and will pay the cost of task switching only when this is really needed [27].

A “meso-scopic” modeling approach, which merges models of different time or space scales, was first suggested for honeybees in [92]. Although this model does not explicitly rely on common stomach regulation, it suggests shared-substance and shared-labor mechanisms in several social insect groups and reconciles them with other mechanisms proposed in literature like “foraging for work” [93, 94] or “threshold-adaptation” [95]. These mechanisms are not necessarily contradicting the common stomach regulation hypothesis but are either augmenting it or emphasizing different points of view of this or a similar regulatory system [29, 96]. In a system regulated by a common stomach, workers have to forage for material (this is also a “work” to do) and the frequencies at which they then change their tasks can be, on average, be interpreted as thresholds [29]. We are confident that the task regulation model based on the governing role of the common stomach in division of labor regulation and in nutrient allocation will contribute as a basic building block to such a future holistic mathematical framework for understanding social insect’s colony integration and self-regulation.

## Supporting information

S1 File Supplemental material. (DOCX)
