# Correction: Resilience of honeybee colonies via common stomach: A model of self-regulation of foraging

**DOI:** 10.1371/journal.pone.0212147

**Published:** 2019-02-06

**Authors:** Thomas Schmickl, Istvan Karsai

Fig 1 is incorrect. Please see the corrected Fig 1 here.

**Fig 1:**
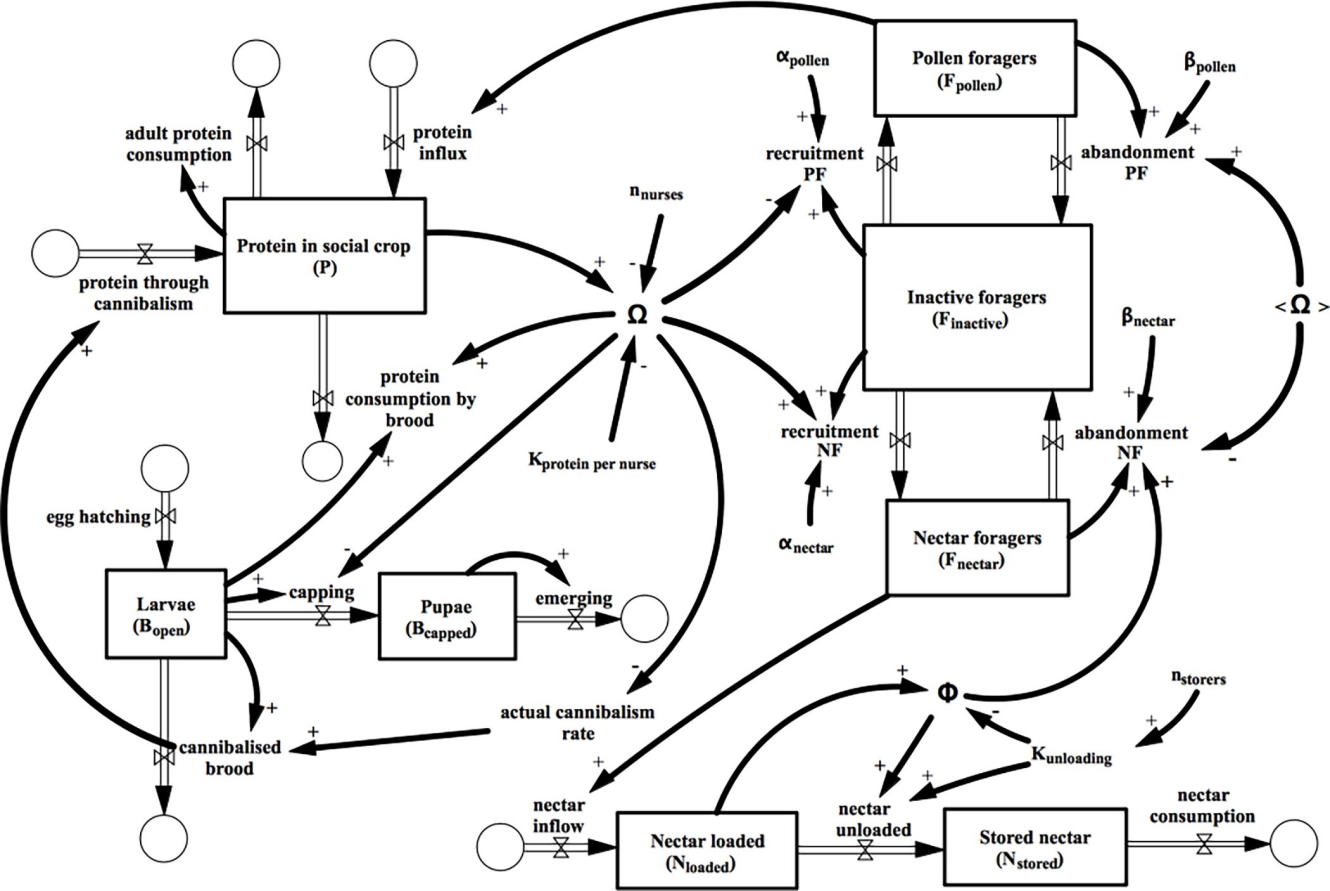
Stock&flow representation of our model system.
