# Supplementary material for: Resilience of honeybee colonies via common stomach: A model of self-regulation of foraging
Source: PLoS One. 2017 Nov 21;12(11):e0188004. doi: 10.1371/journal.pone.0188004 (PMC5697885; doi:10.1371/journal.pone.0188004)
Supplement: S1 File — (DOCX) [file pone.0188004.s001.docx]

## S1 File (Supplemental Material)

## General model building and design principles

In our model building we are following several model design principles described in detail in Schmickl and Karsai (2014). For this model we have extended the list of principles originally proposed by Gilpin and Ayala (1973) as follows:

1. The principle of natural assumptions, which demands that the parameterization is following closely empirical measurements reported from biological studies in literature.
2. The principle of robust processes, which demands that the formulation of the mechanisms ensures stable and plausible performance (e.g., the model does not predict negative population numbers) with the full range of parameter values that are physically possible.
3. The principle of shared substances, which demands that all compartments (such as common stomachs) that contain substances are obeying the physical conservation laws.
4. The principle of local interaction, which demands that all actors (bees, larvae) have a limited sensing and actuation radius, thus there is no central global supervisor unit operating in the modeled system. In consequence, all interactions have to be density and frequency dependent, thus follow the “mass action law”.
5. The principle of simplicity, which demands that the model is kept as simple as possible by including only important factors and mechanisms relevant to our research questions.
6. The principle of universality, which demands that all comparisons of the model to empirical studies are all carried out with the same model. Parameters are only changed in order to accommodate conditions of different experimental setups (e.g., different seasons, different colony sizes, or different foraging conditions).

## Parameters and variables of the model

Our default model parameters reflect an average honeybee colony in early summer. The colony size is set to n_adults_ = 30,000 adult bees (Table A). The initial number of bees available for different tasks are calculated as: n_foragers_ = n_adults_ * 0.4; n_storers_ = n_adults_ * 0.2; n_others_ = n_adults_ * 0.1; n_nurses_ = n_adults_*0.3 (all units: bees). This reflects roughly the fractions of bees in the corresponding age classes that preferentially perform these tasks (Lindauer 1952; Seeley 1982; Sakagami 1953). However, in some simulation experiments we added or removed foragers as a perturbation experiment. In those runs n_foragers_ was turned into a time-dependent variable n_foragers_(t), as the population of foragers changed due to our experimental procedures. Except those perturbation experiments all parameters n_foragers_, n_adults_, n_storers_, n_others_, n_nurses_ were kept constant. However, for our dynamic model, we split up the group of foragers (n_foragers_) into 3 groups that were: pollen foragers (F_pollen_(t)), nectar foragers (F_nectar_(t)) and inactive foragers (F_inactive_(t)), so that F_inactive_(t) = n_foragers_ - F_pollen_(t) - F_nectar_(t). These three system variables can change over time what is described in equations 3-5 in the main text.

The dynamics of the nectar stores are handled in “nunits” with 1 nunit representing one full cropload of nectar volume (54 µl, according to Goyret and Farina (2005)). Protein reserves (in form of pollen stocks and protein shared among the bees) are tracked in “punits” with 1 punit representing the amount of proteins contained in one pollen forager’s load. By default we started the colony in our simulation runs with B_open_(0) = 4,000 larvae, B_capped_(0) = 12,000 pupae, N_loaded_(0) = 10,000 nunits, N_stored_(0) = 1,000,000 nunits (equivalent to 40,000 cells), P(0) = 34,000 punits (equivalent of 2,000 cells). We consider the units “animal”, “bee”, “larvae”, “pupae” and “brood” synonymously, as we have to use those units depending on the context of developmental stage and do not want to overcomplicate the model by introducing specific “conversion factors” of value “1.0”. Given the values above, our model colony has 30,000*0.2 = 6,000 storer bees, representing a maximum unloading capacity of K_unloading_ = 6,000 nunits.

Table A. Parameters and variables used. Details of parameter estimation are described in the text (section “Model parametrization”) and in Schmickl and Karsai (2016) and Schmickl and Crailsheim (2007).

| **Description of stock variables** | **Variable names** | **Initial values** | **Units** |
| --- | --- | --- | --- |
| Pollen forager bees | F_pollen_ | 0.0 | bees |
| Nectar forager bees | F_nectar_ | 0.0 | bees |
| Inactive forager bees | F_inactive_ | 12,000 | bees |
| Open brood | B_open_ | 4,000 | brood |
| Capped brood | B_capped_ | 12,000 | brood |
| Nectar load of all foragers | N_loaded_ | 10,000 | nunits |
| Stored nectar | N_stored_ | 1,000,000 | nunits |
| Protein in common stomach | P | 34,000 | punit |
|  |  |  |  |
| **Description of constants** | **Parameter names** | **Constant values** | **Units** |
| Forager bees | n_foragers_ | 12,000 | bees |
| Storer bees | n_storers_ | 6,000 | bees |
| Nurse bees | n_nurses_ | 9,000 | bees |
| Other bees | n_others_ | 3,000 | bees |
| Abandonment rate of pollen forager | β_pollen_ | 0.25 | 1/hr |
| Recruitment rate of pollen forager | α_pollen_ | 0.15 | 1/hr |
| Abandonment rate of nectar forager | β_nectar_ | 0.25 | 1/hr |
| Recruitment rate of nectar forager | α_nectar_ | 0.5 | 1/hr |
| Nectar collected by a forager | χ_nectar_ | 0.252 | nunits/bee/hr |
| Maximum unloading interval | λ_max_unloading_ | 1.0 | 1/hr |
| Nectar consumption rate of colony | λ_nectar_colony_ | 0.001 | 1/hr |
| Maximum unloading capacity | K_unloading_ | 6,000 | nunits |
| Nectar consumption of larvae | γ_nectar_brood_open_ | 0.02 | nunits/bee/hr |
| Nectar consumption of pupa | γ_nectar_brood_capped_ | 0.005 | nunits/bee/hr |
| Nectar consumed by foragers | γ_foragers_ | 0.021 | nunits/bee/hr |
| Maximum protein saturate a nurse | K_protein_per_nurse_ | 7.0 | punits/bee |
| Protein load | χ_protein_ | 1.0 | punit/bee/hr |
| Nectar load capacity per storer bee | K_nectar_per_storers_ | 1.0 | nunits/bee |
| Base protein consumption of brood | γ_base_feeding_ | 0.08 | punits/bee/hr |
| Reduced consumption of hungry brood | γ_hunger_feeding_ | 0.04 | punits/bee/hr |
| Mortality rate | μ_base_ | 0.00042 | 1/hr |
| Mortality when protein shortage | μ_hunger_ | 0.011 | 1/hr |
| Protein gain from cannibalism | χ_cannibalism_ | 4.833 | punits/larva |
| Colony protein consumption rate | λ_protein_colony_ | 0.005 | 1/hr |
| Eggs hatch to larva | χ_brood_ | 66 | larvae/hr |
| Capping rate in normal conditions | λ_base_capping_ | 0.00757 | 1/hr |
| Capping rate of hungry larva | λ_hunger_capping_ | 0.0083 | 1/hr |
| Emerging rate from pupae to adults | λ_emerging_ | 0.0035 | 1/hr |
| Fraction of time for collecting | τ_foragingfraction_ | 0.5 | dmnl |
|  |  |  |  |
| **System variables** | **Variable names** | **Value range** | **Units** |
| Nectar saturation of the colony (common stomach A) | Φ | 0.0 - 1.0 | dmnl |
| Protein saturation of worker bees (common stomach B) | Ω | 0.0 - 1.0 | dmnl |

## Model parametrization

In the following we describe how we parametrized our model reported in Table A based on either available literature data or on causal deduction. The basic recruitment and abandonment rates were set to values based on the assumption that each forager flies on average 4 times before giving up the foraging and becoming inactive again. However, these basic initial rates change as the common stomach saturations change. The recruitment rates were set to a value that significantly more foragers are recruited to nectar foraging than to pollen foraging. We assumed that inactive foragers resume working on average after a time period between two and eight hours.

We assumed the egg-laying rate of the queen is 1600 eggs/day (Bodenheimer 1937; Schmickl and Crailsheim 2007). We assumed no mortality during the egg phase, thus we assumed the egg-to-larvae hatching rate χ_brood_ = 1,600/24 = 66 larvae/hr. The normal capping rate λ_base_capping_ of larvae was based on a 5.5 day duration of the larvae state, thus we set λ_base_capping_ = 1/(5.5*24) = 0.00757 hr^-1^. The emerging rate of sealed brood λ_emerging_ was based on an average duration of 12 days for the pupal stage (Fukuda and Sakagami 1968), thus we set λ_emerging_ = 1/(12*24) = 0.0035 hr^-1^. In case of protein stress larvae get capped half a day earlier, thus capping is accelerated hence the capping rate λ_hunger_capping_ was set to a higher value of λ_hunger_capping_ = 1/(5.0*24) = 0.0083 hr^-1^ during pollen stress (Schmickl and Crailsheim 2001). The same empirical study (Schmickl and Crailsheim 2001) also showed that a fraction of the larval population gets lost due to brood mortality. This larval mortality was found to increase significantly in times of insufficient protein (pollen) supply, thus we also scaled this loss of larvae as a linear decrease between μ_base_ = 0.00042 hr^-1^ with Ω(t) = 1.0 and μ_hunger_ = 0.011 hr^-1^ with Ω(t) = 0.0 in our model. These values were analyzed in a survivorship model in Schmickl and Crailsheim (2007) showing good agreement with empirical data of Sakagami and Fukuda (1968).

Empirical studies showed that one larva requires 145 mg of pollen (Alfonsus 1933) and 163mg of nectar (Harbo 1993) for normal development. This translates into 145/15 ≈ 10 punits of protein and 163/54 ≈ 3 nunits of nectar to rear one larva. Given that the brood gets fed during 5.5 days (the heating expenses of other brood stages are covered with an extra colony-level consumption rate) we set γ_basefeeding_ 10/(5.5*24) ≈ 0.08 punits/larva/hr and γ_nectar_brood_open_ = 3/(5.5*24) ≈ 0.02 nunits/larva/hr as hourly consumption rates. The heating expenses for the capped brood were assumed to be a small rate of γ_nectar_brood_capped_ = 0.005 nunits/bee/hr. In times of pollen shortage, larvae are fed with less proteinaceous food (Schmickl and Crailsheim 2002; Schmickl et al. 2003). We set the reduced protein consumption (50% of the normal level) rate of larvae to γ_hungerfeeding_ = 0.04 punits/larva/hr.

The parameter K_protein_per_nurse_ = 7 punits/bee represents the maximum protein that can saturate one nurse. The value of K_protein_per_nurse_ is based on the following deduction: A full-grown colony of 30,000 bees can have approximately 8,000–10,000 nurse bees, based on the fact that the nursing task is performed for about 8-10 days in the lifetime (Seeley 1982). An average colony will have pollen reserves of approximately 3,500 pollen cells (Jeffree and Allen 1956) and each contains approximately 230 mg of pollen (Camazine et al. 1990). In consequence, 1 punit represents 0.065 cells of pollen, and thus 3,500 pollen cells for 8,000-10,000 nurse bees represent saturation levels between 5 and 7 punits per nurse bee.

Honeybees can regain a fraction of proteins, which were invested into these larvae before, by cannibalizing them (Schmickl and Crailsheim 2001; 2004; Webster and Peng 1987; Webster et al. 1987). We assumed that only 50% of the proteins can be regained, thus we set the regaining rate to χ_cannibalism_ = (145*0.5)/15 = 4.833 punits/larva, assuming that a larva is fully consumed in our model within 1h. Furthermore, we assumed steady consumption rates λ_proteincolony_ = 0.005 hr^-1^ and λ_nectar_colony_ = 0.001 hr^-1^, representing the fractions of protein and nectar stores consumed by all bees in the colony per hour for processes which were not explicitly modeled in our paper. This assumption was based on the observation that stored pollen is consumed at a much higher rate than stored honey, causing faster honey accumulation (Camazine et al. 2003).

The average pollen foraging flight yields 15mg of collected pollen while the average nectar foraging trip is 30 mg/flight (Seeley 1995). Huang and Seeley (2004) reports 12.6 µl/flight nectar inflow as low boundary and 17.0 µl/flight nectar inflow as high boundary of natural nectar influx. Omholt (1992) reported 15 mg/flight nectar influx. Based on these empirical data we decided that one foraging trip results in χ_nectar_ = 13.6/54 = 0.252 nunits/bee/hr in our model and every pollen trip equals 1 punit/bee/hr what corresponds to 15mg of collected pollen on average. Foraging flight have energetic costs, thus we assumed a rate of γ_foragers_ = 0.021 nunits/bee/hr cost for both types of foraging flights. This represents a cost-to-gain ratio of approx. 8%, what is a plausible range for a natural foraging flight (flying from flower to flower). This value is higher than the 2% reported by Seeley (1994), but in his experiment the foraging flights targeted artificial feeders which allow the bees to fill up their crops with nectar by visiting just a single location.

By definition, the protein content of a bee’s foraging trip was set to χ_protein_ = 1.0 punits/bee/hr, as this is the way how we defined the unit of protein collection trip punit. Following the same reasoning, the maximum nectar load of a storer bee was set to K_nectar_ = 1.0 nunits/bee and the maximum unloading interval λ_max_unloading_ = 1.0 hr^-1^, (our model operates on time steps of 1hr). Our model does not discriminate between day and night, thus we set τ_foragingfraction_ = 0.5 to adjust the foraging in our model to a foraging period of 12 hours per day.

## Modeling the unloading process

The saturation of nectar-storing bees is a cornerstone element in the functioning of the common stomach Φ(t). We considered three alternatives to model the unloading process (Fig. A) and ultimately selected the best one for our unloading function.

1.) Model 1 (dashed line in Fig. A) assumes that the unloaded nectar (nectar_unloaded(t)) was equal to the amount of nectar brought to the colony by nectar foragers (N_loaded_(t)), thus it assumed that every returned forager gets unloaded within a time step. In this model, the common stomach can never be saturated. Although this is the simplest function that can be used, we discarded this formulation, because it violates our model building “principle of natural assumptions”.

2.) Model 2 (dotted line in Fig. A) assumes a formulation that levels off the unloaded nectar with the unloading capacity (K_unloading_) by using a “min()”-function. This method was used already in (Karsai and Schmickl 2011; Karsai and Phillips 2012; Hamann et al. 2013) for modeling the water unloading of loaded water foragers in paper wasps. This formulation leads to a linear increase until N_loaded_(t) reaches K_unloading_, then it makes a sudden edge after which the level of the flow nectar_unloaded(t) stayes exactly at the level of K_unloading_. This approach is simple and addresses the fact that the common stomach has limited capacity, however a recent detailed model of the functioning of the common stomach (Agrawal and Karsai 2016) revealed that the common stomach starts to “resist” to be filled up even before it is full. Therefore, we can assume that the unloading efficiency decreases with increasing saturation of the nectar storage workforce already long before this common stomach is fully saturated. Thus model 2 was discarded for the benefit of a new formulation (model 3), which considered the mass-action law for describing the interaction of food storers with loaded nectar foragers in a more natural way and which better fits to our “principle of shared substance”.

3.) Model 3 (solid line in Fig. A) assumes that the interaction between food storer bees and nectar foragers which are to be unloaded is following the classical mass-action law, which is given by N_loaded_(t)*K_unloading_. From this interaction term the model for the unloading of nectar has to consider the common stomach Φ(t) = N_loaded_(t)/(N_loaded_(t) + K_unloading_). This ultimately yields the final model for the dynamics of the unloaded nectar amount per time step: nectar_unloaded(t) = Φ(t)*K_unloading_. This model is consistent with all our model-building principles as it is built on natural assumptions, it describes a robust process because it is part of a negative feedback loop, it models a shared substance correctly by mass-action law, it assumes only local interaction, it is based on a simple and known universal law of scientific disciplines. Thus, we adopted this formulation for our model.

Fig. A. Three different models to predict the unloading process. Dashed line (model 1) assumes no limitation by storer bees. Dotted line (model 2) assumes a limitation by storer bees, but this limitation suddenly jumps into action as soon as foragers return more nectar than can be handled. The solid line (model 3) shows the model actually used, which assumes a slow saturation of the common stomach of nectar storers as the amount of collected nectar increases. For the exemplary case shown here, K_unloading_ is assumed to be 1,000 nectar units per hour.

## References

Agrawal D, Karsai I. The mechanisms of water exchange: The regulatory roles of multiple interactions in social wasps. PLoSONE 2016; 11(1): e0145560. doi:10.1371/journal.pone.0145560.]

Alfonsus EC. Zum Pollenverbrauch des Bienenvolkes. Arch. f. Bienenk. 1933; 14: 220–223.

Bodenheimer FS. Studies in animal populations II. Seasonal population-trends in the honey-bee. Quat. Rev. Zool. 1937, 12: 406–425.

Camazine S, Deneubourg JL, Franks NR, Sneyd J, Theraulaz G, Bonabeau E. Self-Organization in Biological Systems. Princeton University Press. 2003; pp. 560. SBN: 9780691116242.

Camazine S, Sneyd J, Jenkins J, Murray JD. A mathematical model of self- organized pattern formation on the combs of honeybee colonies. J. Theor. Biol. 1990; 147: 553–571.

Fukuda H, Sakagami SF. Worker brood survival in honey bees. Res. Popul. Ecol. 1968; 10: 31–39.

Gilpin ME, Ayala FJ. Global models of growth and competition. Proc. Natl. Acad. Sci. USA. 1973; 70: 3590-3593.

Goyret J, Farina WM. Trophallactic chains in honeybees: a quantitative approach of the nectar circulation amongst workers. Apidologie. 2005; 36: 595–600.

Hamann H, Karsai I, Schmickl T. Time delay implies cost on task switching: a model to investigate the efficiency of task partitioning. B. Math. Biol. 2013; 75: 1181-1206. DOI 10.1007/s11538-013-9851-4.

Harbo JR. Effect of brood rearing on honey consumption and the survival of worker honey bees. J. Apic. Res. 1993; 32: 11–17.

Huang MH, Seeley TD. Multiple unloadings by nectar foragers in honey bees: a matter of information improvement of crop fullness? Insectes Soc. 2004; 50: 330–339.

Jeffree EP, Allen MD. The annual cycle of pollen storage by honey bees. J. Econ. Entomol. 1956; 50: 211–212.

Karsai I, Phillips MD. Regulation of task differentiation in wasp societies: A bottom-up model of the “common stomach”. J. Theor. Biol. 2012; 294: 98-113.

Karsai I, Schmickl T. Regulation of task partitioning by a ''common stomach'': a model of nest construction in social wasps. Behav. Ecol. 2011; 22: 819–830.

Lindauer M. Ein Beitrag zur Frage der Arbeitsteilung im Bienenstaat. Z. Vergl. Physiol. 1952; 34: 299–345.

Omholt S. The heuristic value of mathematical modelling for elucidation of the honey production dynamics of *Apis mellifera* colonies. Norw. J. Agric. Sci. 1992; 6 (2): 99–110.

Sakagami SF. Untersuchungen über die Arbeitsteilung in einem Zwergvolk der Honigbiene. Beiträge zur Biologie des Bienenvolkes, *Apis Mellifera* L.I. Jpn. J. Zool.1953; 11: 117–185.

Sakagami SF, Fukuda H. Life tables for worker honeybees. Res. Popul. Ecol. 1968; 10: 127–139.

Schmickl T, Crailsheim K. HoPoMo: A model of honeybee intracolonial population dynamics and resource management. Ecol. Model. 2007; 204: 219–245.

Schmickl T, Karsai I. How regulation based on a common stomach leads to economic optimization of honeybee foraging. J. Theor. Biol. 2016; 389: 274-286.

Schmickl T, Karsai I. Sting, Carry and Stock: How Corpse Availability Can Regulate De-Centralized Task Allocation in a Ponerine Ant Colony. PLoS ONE 2014; 9(12): e114611. doi:10.1371/journal.pone.0114611.

Schmickl T, Blaschon B, Gurmann B, Crailsheim K. Collective and individual nursing investment in the queen and in young and old honeybee larvae during foraging and non-foraging periods. Insectes Soc. 2003; 50: 174–184.

Schmickl T, Crailsheim K. Cannibalism and early capping: strategies of honeybee colonies in times of experimental pollen shortages. J. Comp. Physiol. A 2001; 187: 541–547.

Schmickl T, Crailsheim K. How honeybees (*Apis mellifera* L.) change their broodcare behaviour in response to non-foraging conditions and poor pollen conditions. Behav. Ecol. Sociobiol. 2002; 51: 415–425.

Seeley TD. Honey bee foragers as sensory units of their colonies. Behav. Ecol. Sociobiol. 1994; 34: 51-62.

Seeley TD. Adaptive significance of the age polyethism schedule in honeybee colonies. Behav. Ecol. Sociobiol. 1982; 11: 287–293.

Seeley TD. The Wisdom of the Hive: The Social Physiology of Honey Bee Colonies. Harvard University Press, Cambridge/Massachusetts, 1995; pp. 368.

Webster TC, Peng Y-S. Passage of cannibalized tissue among honey bee (Hymenoptera: Apidae) colony members. Ann. Entomol. Soc. Am. 1987; 80: 814–819.

Webster TC, Peng Y-S, Duffey SS. Conservation of nutrients in larval tissue by cannibalizing honey bees. Physiol. Entomol. 1987;12: 225–231.
